# Non-covalent interactions from a Quantum Chemical Topology perspective

**DOI:** 10.1007/s00894-022-05188-7

**Published:** 2022-08-25

**Authors:** Paul L. A. Popelier

**Affiliations:** Department of Chemistry, University of Manchester, Oxford Road, Manchester, M13 9PL Great Britain UK

**Keywords:** Quantum Chemical Topology (QCT), Relative Energy Gradient (REG), FFLUX, QTAIM, Interacting Quantum Atoms (IQA), Atomic electron correlation, Polarisation, Machine learning, Gaussian process regression, Multipole moments, Exchange energy, Covalency

## Abstract

About half a century after its little-known beginnings, the quantum topological approach called QTAIM has grown into a widespread, but still not mainstream, methodology of interpretational quantum chemistry. Although often confused in textbooks with yet another population analysis, be it perhaps an elegant but somewhat esoteric one, QTAIM has been enriched with about a dozen other research areas sharing its main mathematical language, such as Interacting Quantum Atoms (IQA) or Electron Localisation Function (ELF), to form an overarching approach called Quantum Chemical Topology (QCT). Instead of reviewing the latter’s role in understanding non-covalent interactions, we propose a number of ideas emerging from the full consequences of the space-filling nature of topological atoms, and discuss how they (will) impact on interatomic interactions, including non-covalent ones. The architecture of a force field called FFLUX, which is based on these ideas, is outlined. A new method called Relative Energy Gradient (REG) is put forward, which is able, by computation, to detect which fragments of a given molecular assembly govern the energetic behaviour of this whole assembly. This method can offer insight into the typical balance of competing atomic energies both in covalent and non-covalent case studies. A brief discussion on so-called bond critical points is given, highlighting concerns about their meaning, mainly in the arena of non-covalent interactions.

## 1 Introduction

While chemistry used to be mainly the science of the molecule, much of this central discipline has moved on to become the science of the molecular assembly. This part of Nature is governed by so-called non-covalent interactions, which are energetically weaker than covalent interactions. This weakness poses a challenge on two fronts: quantitative computation and qualitative interpretation. On the one hand, the quantitative front is conveniently illustrated by the fact that, so far, no computation has ever predicted a successful drug. Certainly, the energetics of drug design is one where each kilojoule per mole matters in the calculation of protein–ligand interaction. On the other hand, a spectacular example of the qualitative front is the hypothesis [1] that a gecko’s ability to defy gravity is due to attractive van der Waals forces in hairs on its feet. The difficulty here was that later work discredited [2] this interpretation.

Looking at the totality of the “non-covalent literature”, including all scales (from noble gas dimers to proteins in aqueous solution and beyond) and all methods (first principles and force fields), reveals many successes. Yet, there is currently no fully integrated and consistent numerical treatment (i.e. quantitative prediction) of non-covalent interactions across all system scales and research fields. Secondly, interpretative models used by experimentalists often remain too simple, and poorly connected to a rigorous (quantum) physical reality. In summary, it will probably take a long time before scientists meet the challenge of both quantitatively predicting and qualitatively understanding non-covalent interactions.

The importance of properly grasping non-covalent interaction energies and forces cannot be underestimated. One can only marvel at the complexity of the meticulous packing of biomolecules in a living cell. For example, inside the nucleus, each chromosome contains a long DNA double helix, which is cleverly wrapped around proteins called histones. Again, non-covalent interactions govern this apparently delicate yet robust process, not to mention the stability of the double helix itself. Even more stunning is the complexity of transcription in the nucleus. The enzyme RNA polymerase attaches to a gene (a piece of DNA) and starts making messenger RNA, steered by molecular complementarity. This mechanism, as well as the architecture and operation of this whole molecular machine, is again managed by non-covalent interactions. That such an ingenious process automatically materialised in Nature seems to suggest an as of yet unstated law; something within irreversible thermodynamics just has to lead to such wonders of organisation. Other biochemical processes are even more breath-taking in their complexity and precision, to the point that one begins to question how all this can actually emerge from the non-covalent forces that quantum chemists hope to master. Will, in a century perhaps, embryology become a special branch of chemistry?

In this article, much more down-to-earth aspects of non-covalent interactions will be discussed from the point of view of Quantum Chemical Topology (QCT), [3–6] which is a generalisation of the Quantum Theory of Atoms in Molecules (QTAIM) [7–10]. There are a number of directions that this article will not follow because they have been reviewed elsewhere or do not meet its intent and spirit. One such direction is teaching technical content behind QCT because several pedagogical accounts (see the four references on QCT mentioned just above) have done so recently. The reader is kindly requested to consult those references because this Conversation is unfortunately not a tutorial. Secondly, the direction of reviewing [11] the by now vast literature that uses the original [10, 12] QTAIM descriptors (to characterise all sorts of atomic interactions across all areas of chemistry) will not be followed either. Many such studies, such as a fairly recent one [13] on E…π (E = O, S, Se, and Te) and E…π interactions, report only local properties evaluated at so-called bond critical points (see Section 2.11). There is also interesting quantum topological work on halogen bonding, for example, that has again been surveyed [14] elsewhere. Another important branch of QCT is the richly documented topology [15] of the so-called Electron Localisation Function (ELF). This function too has been used in the study of non-covalent interactions (see reference [16] for a recent example) but again this noteworthy work falls outside the scope of this paper. What is then the direction followed? The bias of this article is on integrated properties (such as charges, higher rank multipole moments, and energies). A number of rather bold statements (listed in subsection of the “Proposals and claims” of Section 2) will be made, with an eye on complying with the intent and character of this type of article.

## 2 Proposals and claims

### 2.1 How to define an atom inside a system?

**Fig. 1:**
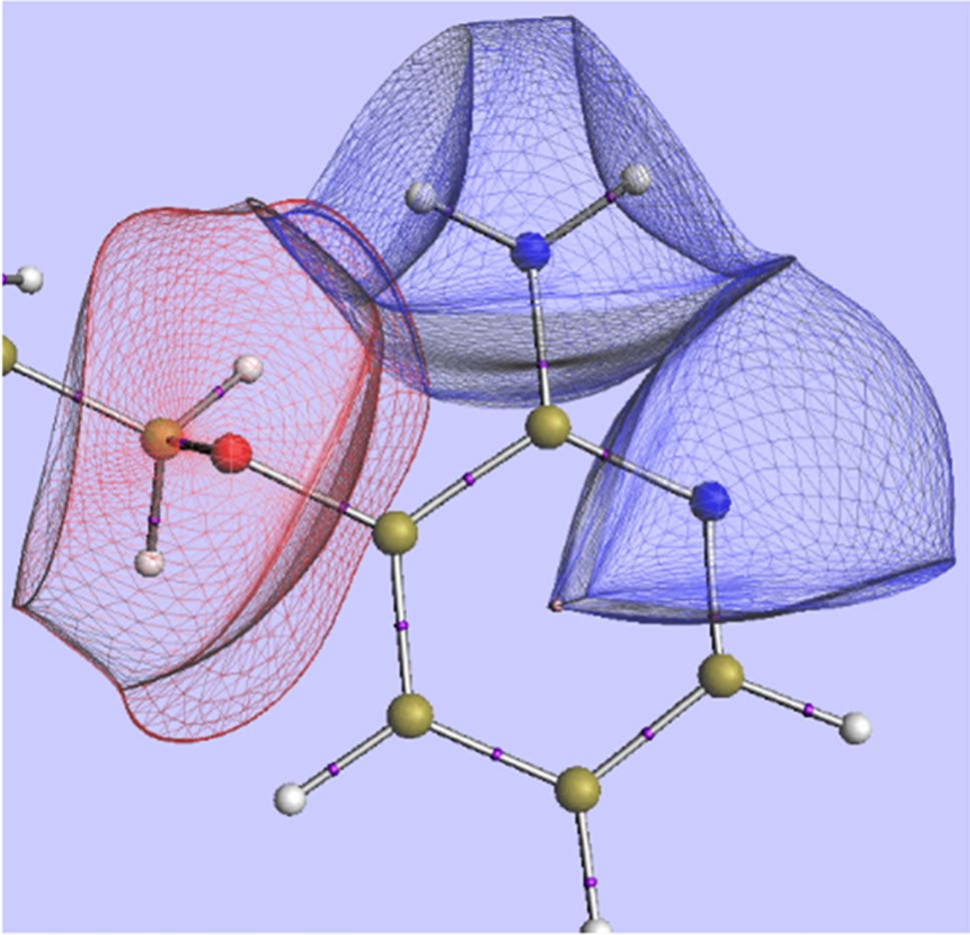
Examples of topological atoms: an ether oxygen, a pyridine nitrogen, and an amine nitrogen

Many will agree that non-covalent interactions are typically described at atomistic level except perhaps for an elementary description of π-π stacking where the *molecular* quadrupole moments of, for example, substituted benzenes are brought in. Thus, there is a need to define an atom within a molecule (or molecular assembly). How to do this is a contentious question, which cannot be reviewed here due to space restriction. However, five arguments in favour of the quantum topological proposal can be rehearsed here. To set the scene, Fig. 1 shows examples of topological atoms.

A first argument is that the topological approach takes the (molecular) electron density as its starting point rather than a Hilbert space of basis functions. The topological partitioning is therefore a real space method that operates in ordinary 3D space. This feature has two advantages. Firstly, the definition of the atom survives upon varying the generator of the electron density (e.g. SCF-LCAO-MO, X-ray crystallography, or a grid-based computational scheme). In other words, the topological atom does not depend on the details of how the electron density was obtained. Topological atoms transcend orbitals; their shapes do not depend on any choice made here. Because the electron density is observable, atomic properties can be obtained from both theory and experiment. The second advantage is that atomic charges are robust with respect to the nature of the basis set. To the contrary, the concept of Mulliken charges, for example, becomes unstable in the presence of diffuse Gaussian primitive functions and even evaporates altogether when using plane-wavefunctions. Similarly, the well-known Distributed Multipole Analysis (DMA) [17] leads to atomic charges that become unreliable when diffuse Gaussian primitives are used. However, this problem has been overcome [18] by essentially introducing less fuzzy boundaries to the DMA atoms. This modification acknowledges the benefit offered by sharper boundaries, which also underpin the QTAIM philosophy. We note that DMA lies at the heart of a number of next-generation multipolar force fields such as SIBFA, [19] XED, [20] EFP, [21] AMOEBA, [22] NEMO, [23] and the force field behind the crystal structure prediction code DMACRYS, [24] which will hopefully replace classical force fields such as AMBER, CHARMM, OPLS, MM3, or GROMOS. Thorough comparisons [25, 26] between 7 Hirshfeld variants of atomic charge, the QTAIM charge, and 4 ESP-fitted types of charges show that QTAIM is the most robust, i.e. not too sensitive to details in the electronic structure calculations from which they are derived (basis set, conformational changes, chemical changes in the environment).

A second argument in favour of the topological approach is that it is minimal (not to be confused with “simple” (see preface of ref [27])) in the sense of Occam’s razor. The only notion needed to reveal the atoms is the gradient path, which is a trajectory that always follows the direction of maximal ascent of the function at hand (i.e. the electron density). This notion is sufficient to recover the language of dynamical systems and algebraic topology (p. 31/32 in ref [6]): atomic basins (i.e. topological atoms), critical points, separatrices (i.e. interatomic surfaces), conflict and bifurcation catastrophes, the gradient vector field, critical points, and attractors. The concept of the gradient path is parameter-free other than for practical decisions (i.e. numerical method and settings) on how to solve the system of differential equations that generate the gradient paths. Moreover, no reference density is brought in, unlike in the case of high-resolution crystallographic deformation densities or Hirshfeld charges, for example. We note that the original Hirshfeld approach [28] was made independent of its damaging promolecular reference density, resulting in the so-called Hirshfeld-I method [29]. Interestingly, the new atomic charges thus obtained become larger in magnitude than the original ones. Thus, the iterative Hirshfeld charges moved towards the topological charges, which were ironically often criticised for being too large. The further developments around Hirshfeld charges have more problems and subsequent solutions but they are beyond the scope of this article. Finally, we note that, in addition to Hirshfeld-I, the MBIS and ISA approaches also do not have the problems shown by the original Hirshfeld approach.

Thirdly, within the real space partitioning approach, there are fuzzy (i.e. interpenetrating) or non-fuzzy (i.e. space-filling) methods. QCT belongs to the latter category because topological atoms *do not overlap nor do they leave gaps between them*. A thorough and systematic comparison [30] of both types of partitioning concluded that fuzzy partitions give small atomic net charges and enhanced covalency, while space-filling partitions generate larger net charges and smaller covalencies. The smallest deformations respective to a reference are found in space-filling decompositions, which generate a less distorted image of chemical phenomena leading to smaller deformation and interaction energies. This is an important conclusion because it is at the heart of chemistry: space-filling decompositions better preserve the atomic (or fragment) identity from the energetic point of view.

Fourthly, from Gauss’s divergence theorem, it can be proven that a topological atom has a well-defined kinetic energy. Put differently, an arbitrary subspace would suffer from an ambiguous kinetic energy. There are at least two different ways to define a local kinetic energy density, and for an arbitrary subspace, they each return a different kinetic energy. However, for a topological atom, the two energies are the same. An atom with such a well-defined kinetic energy is called a quantum atom. Note that all topological atoms are quantum atoms but not vice versa. When integrating over the special (zero-flux) volume that is a topological atom, the otherwise digressing kinetic energies (for an arbitrary volume) congregate to the same value. However, Anderson et al. [31] showed that all this is only true within what they call the “Laplacian family of local kinetic energies” when introducing quasiprobability distributions. But then again, this expanded knowledge does not reduce [32] the value of the topological atom; it just points out that not all quantum atoms are topological atoms.

Fifthly and finally, a significant effort was made to answer the question if quantum mechanics can provide a complete description of an atomic subsystem. Schwinger’s principle [33] served as a starting point to answer this question because of the elegance of its unified approach: as a single principle, it provides a complete development of quantum mechanics. The answer to the question posed above is affirmative because the same principle applies [34] to an atomic subsystem when it is a topological atom. However, it was shown [35] much later that the topological atom does not uniquely follow from quantum mechanics as the only quantum atom.

The advantages listed so far need to be balanced by the disadvantage of computational expense and algorithmic difficulties that QCT introduces. A brief literature review on QCT algorithms has been given before, in both Introductions of two previous papers [36, 37] on new algorithms. The computational expense of calculating atomic properties has slackened the uptake of QCT over the decades but this infelicity dwindles by the year through the automatic advent of improved hardware. Furthermore, improved algorithms cope better with the complexity of delineating the boundary of a topological atom as seen by an integration ray (centred at the nucleus) while sweeping the atom’s volume. However, setting up a completely robust and efficient algorithm remains a challenge.

We end this section with a quasi-philosophical comment on the nature of space-filling atoms. The mainstream view on the nature of atoms inside molecules appears to be one that regards atoms as fuzzy, interpenetrating objects. This view then also applies to the molecules that atoms form: molecules too will penetrate each other and thus overlap. We will return to this point in Section 2.4, with the alternative view following from QCT. A central question is, looking at the natural world, can one find support for the idea of non-overlapping objects? Life itself could not have emerged were it not for its sharp boundaries, starting with cells and the many confined, membraned organelles within them. At a conceptual level, there are several more important examples: the phases in a phase diagram do not overlap, and thermodynamics exhibits a sharp distinction between the system and the surroundings. To push examples further, beyond the physical sciences: Portugal and Spain do not overlap, and legally one is dead or alive, or married or not.

### 2.2 Quantum topological energy partitioning

The birth of QTAIM, now almost half a century ago, can be traced back to a paper published [38] in 1972, which for the first time showed the emblematic shape of an interatomic surface. The authors showed the existence of an atomic subspace obeying its own virial relationship and illustrated this for a number of lithium-containing diatomics. The follow-up paper [39] showed that the total energy of a topological atom can be obtained without calculating its potential energy thanks to the existence of the atomic virial theorem. However, this is only possible if the forces on all the nuclei in the molecule (that the atom is part of) vanish. The fact that atomic energies could only be computed for a molecule at a stationary point on its potential energy surface remained an undesirable restriction for decades. However, in 1997 an algorithm appeared [40] that calculated the (electrostatic) potential energy between two topological atoms. Unwittingly this work paved the way to break free from the constraint of the virial theorem. In 2001, Salvador et al. [41] and ourselves [42] established an algorithm to calculate interatomic electrostatic potential energies, independently of each other, and both independently of that 1997 paper. This work inspired another group to create their own version 3 years later, [43] which then led to the main paper [44] establishing the IQA method. IQA attracts a growing number of users and supporters as recently reviewed. [45]

Today, IQA is the most used topological energy partitioning method although an alternative one exists [46–48]. IQA enables the calculation of both intra- and interatomic energies for any molecular geometry. Importantly, it also achieves this for non-stationary points on the potential energy surface, which was not possible with the original QTAIM approach. The water hexamer, for example, has been analysed [49] in terms of hydrogen bond cooperativity and anti-cooperativity. More on this work will be mentioned near the end of this section.

Two IQA alternatives exist when combined with DFT: the first one, [50] which is implemented in the popular and fast program AIMAll, [51] and one that followed shortly after [52]. Recent work [53] compared these two energy partitionings with the original virial-based approach. The current variation in topological energy partitionings is much smaller than that in non-topological ones [54].

The brief history above merely serves to put QTAIM in a context, and definitely one that shows that its origin lies in energy partitioning. Indeed, quite often, QTAIM is introduced and portrayed [55] as a population analysis [56] but this is not doing it justice. In fact, it would be more exciting and useful to point out that QTAIM offers both atomic charges and atomic energies from the same underlying idea. This idea is the integration, over an atomic volume, of relevant quantum mechanical property densities, which produce all atomic properties (including volumes and multipole moments). Such universality cannot be claimed by a slightly more recent (1976) and popular energy partitioning scheme, [57] namely that of Kitaura and Morokuma. This scheme offers no corresponding atomic charges.

We now briefly explain how IQA partitions the total energy of a system, *E*_*tot*_, which can be written as follows:

1 $${E}_{tot}={E}_{e}+{V}_{nn}={\int {d\mathbf{r}}_{1}(\widehat{T}+{\widehat{V}}_{ne}){\rho }_{1}({\mathbf{r}}_{1};{\mathbf{r}}_{1}^{\boldsymbol{^{\prime}}})|}_{{\mathbf{r}}_{1}^{\boldsymbol{^{\prime}}}\to {\mathbf{r}}_{1}}+\frac{1}{2}\int {d\mathbf{r}}_{1}\int {d\mathbf{r}}_{2}\frac{{\rho }_{2}({\mathbf{r}}_{1},{\mathbf{r}}_{2})\boldsymbol{ }}{{r}_{12}}+{V}_{nn}$$

where ${\rho }_{1}({\mathbf{r}}_{1},{\mathbf{r}}_{1}^{\boldsymbol{^{\prime}}})$ is the non-diagonal first-order reduced density matrix, ${\rho }_{2}({\mathbf{r}}_{1},{\mathbf{r}}_{2})$ the diagonal second-order reduced density matrix, and *V*_*nn*_ the internuclear repulsion energy. Note that **r**_**1**_′ is set to **r**_**1**_ after the Laplacian operator in the kinetic energy operator has acted on **r**_**1**_ only. The one-electron operators $\widehat{T}$ and ${\widehat{V}}_{ne}$ respectively represent the electronic kinetic energy and the attractive nuclear-electron potential energy while the two-electron operator r_12_ expresses the interelectronic repulsion. The integrations take place over the whole of three-dimensional space. However, the topological atomic partitioning introduces integration over atomic volumes Ω_A_ and Ω_B_. For example, the nuclear-electron potential energy between the molecular electron density within Ω_A_ and the nuclear charge of Ω_B_ is given by

2 $${V}_{en}^{AB}=\underset{{\Omega }_{A}}{\overset{}{\int }}{d\mathbf{r}}_{1}{\widehat{V}}_{en}^{B}{\rho }_{1}\left({\mathbf{r}}_{1};{\mathbf{r}}_{1}^{^{\prime}}\right)=-{Z}_{B}\underset{{\Omega }_{A}}{\overset{}{\int }}{d\mathbf{r}}_{1}\frac{\rho ({\mathbf{r}}_{1})}{{r}_{1B}}$$

where r_1B_ is the distance between the nucleus in Ω_B_ and an electron. Similarly, the intra-atomic electron–electron repulsion energy within Ω_A_ is defined by

3 $${V}_{ee}^{AA}=\frac{1}{2}\underset{{\Omega }_{A}}{\overset{}{\int }}{d\mathbf{r}}_{1}\underset{{\Omega }_{A}}{\overset{}{\int }}{d\mathbf{r}}_{2}\frac{{\rho }_{2}\left({\mathbf{r}}_{1}, {\mathbf{r}}_{2}\right)}{{r}_{12}}$$

The mono-atomic energy contributions (also called [42] self-energy) are collected in a single contribution for Ω_A_,

4 $${E}_{intra}^{A}={T}^{A}+{V}_{en}^{AA}+{V}_{ee}^{AA}$$

where T^A^ is the atomic kinetic energy and ${V}_{en}^{AA}$ the nuclear-electron potential energy between Ω_A_’s own nucleus and the molecular electron density that is within this atom’s volume. The overall intra-atomic energy ${E}_{intra}^{A}$ has been fitted successfully to the repulsive part of the Buckingham potential for van der Waals complexes, [58] and more general work [59] of this type also confirms that this IQA term represents steric energy.

The interatomic interaction energies are obtained by invoking the fine-structure of ${\rho }_{2}\left({\mathbf{r}}_{1}, {\mathbf{r}}_{2}\right)$, which involves three well-known contributions: Coulomb (C), exchange (X), and correlation (c). The latter two energies are often combined in one term of representing exchange–correlation (Xc). Each of the three terms that make up ${\rho }_{2}\left({\mathbf{r}}_{1}, {\mathbf{r}}_{2}\right)$ corresponds to an energy, as shown in Eq. (5). This is formally expressed for a pair of interacting atoms Ω_A_ and Ω_B_ as

5 $${V}_{ee}^{AB}=\underset{{\Omega }_{A}}{\overset{}{\int }}{d\mathbf{r}}_{1}\underset{{\Omega }_{B}}{\overset{}{\int }}{d\mathbf{r}}_{2}\frac{{\rho }_{2}\left({\mathbf{r}}_{1}, {\mathbf{r}}_{2}\right)}{{r}_{12}}=\underset{{\Omega }_{A}}{\overset{}{\int }}{d\mathbf{r}}_{1}\underset{{\Omega }_{B}}{\overset{}{\int }}{d\mathbf{r}}_{2}\frac{\rho \left({\mathbf{r}}_{1})\rho {(\mathbf{r}}_{2}\right)}{{r}_{12}}-\underset{{\Omega }_{A}}{\overset{}{\int }}{d\mathbf{r}}_{1}\underset{{\Omega }_{B}}{\overset{}{\int }}{d\mathbf{r}}_{2}\frac{{\rho }_{1}\left({\mathbf{r}}_{1}, {\mathbf{r}}_{2}\right){\rho }_{1}\left({\mathbf{r}}_{2}, {\mathbf{r}}_{1}\right)}{{r}_{12}}+\underset{{\Omega }_{A}}{\overset{}{\int }}{d\mathbf{r}}_{1}\underset{{\Omega }_{B}}{\overset{}{\int }}{d\mathbf{r}}_{2}\frac{{\rho }_{2}^{corr}\left({\mathbf{r}}_{1}, {\mathbf{r}}_{2}\right)}{{\mathbf{r}}_{12}}={V}_{ee,C}^{AB}+{V}_{ee,X}^{AB}+{V}_{ee,c}^{AB}={V}_{ee,C}^{AB}+{V}_{ee,Xc}^{AB}$$

**Fig. 2:**
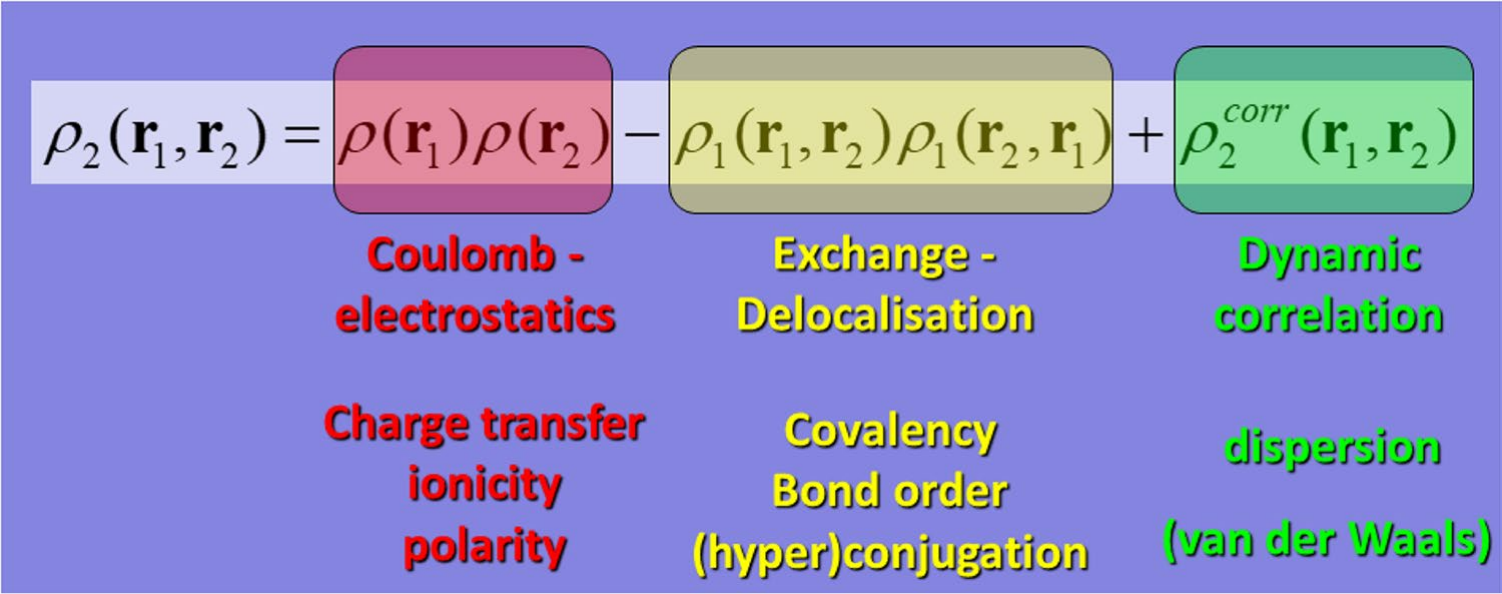
The fine-structure of the second-order reduced matrix alongside the specific chemical insight that each of the three contributions offer

The six-dimensional integrations that are at the heart of the three types of energy contributions are time-consuming but can be carried out by the thousands, on the typical multi-core hardware that most labs have nowadays. Figure 2 illustrates the fine-structure of ${\rho }_{2}\left({\mathbf{r}}_{1}, {\mathbf{r}}_{2}\right)$ and how it relates to various chemical concepts.

A traditional IQA analysis, which operates at atomic resolution and which sees both intra- and interatomic energies, can be generalised. Firstly, due to the space-filling and thus additive nature of topological atoms, it is trivial to obtain intra-group and inter-group energies by simply summing the energies of the participating atoms. A group can be any bunch of atoms, such as a functional group, but it can also be a molecule inside an assembly of molecules. Secondly, it is possible [49] to lump the intra-group (deformation) energy into the inter-group energy. This is no unique way of doing this but a popular choice is using the ratio of an inter-group energy to the sum of all inter-group energies as a weight that contributes to the intra-group energy. As an example, the various configurations of the water hexamer (ring, prism, cage, book, bag) can then be analysed by IQA interaction energies defined for any monomer inside the hexamer. This value is on average − 39 kJ mol^−1^ between any two adjacent monomers in the so-called homodromic ring. This energy stabilises to − 41 kJ mol^−1^ for two waters in the book configuration, where one water forms a hydrogen bond with another water that acts as a double hydrogen acceptor. This subtle hydrogen bond strengthening effect occurs in the presence of the latter’s anti-cooperative effect and came as a surprise.

It is helpful to further illustrate IQA with energies occurring in a wider set of hydrogen-bonded systems. An analysis of 9 simple hydrogen-bonded complexes [60] focuses on groups of atoms, namely the molecules (monomers) in each complex. Before we can discuss the data in Table 1, we need to define a few more energies derived from the primary IQA energies already defined above. Firstly, the mono-atomic energy ${E}_{intra}^{\Omega }$ has been identified with steric energy [58, 59]. It is informative to compare it with the energy of a free atom Ω and thus define the resulting deformation energy as

**Table 1:** IQF analysis (in kJ/mol) and charge transfer (in e) of 9 simple hydrogen-bonded complexes (proton donor, *D*, and proton acceptor, *A*) geometry-optimised at CAS/6–311 + G(d,p) level

Systems		Energies								Charge transfer
Donor (D)	Acceptor (A)	${E}_{\mathrm{def}}^{D}$	${E}_{\mathrm{def}}^{A}$	${V}_{elec}^{DA}$	${V}_{Xc}^{DA}$	${E}_{\mathrm{int}}^{DA}$	${E}_{bind}$	${V}_{elec}^{HB}$	${V}_{Xc}^{HB}$	Q^D^
HF	HF	18.8	22.2	− 20.5	− 38.1	− 58.6	− 17.6	− 344.9	− 21.3	− 0.005
HF	H_2_O	26.8	34.7	− 36.5	− 60.7	− 97.2	− 35.7	− 635.9	− 30.6	− 0.021
HF	NH_3_	36.4	51.9	− 47.3	− 89.2	− 136.5	− 48.2	− 621.6	− 44.0	− 0.050
H_2_O	H_2_O	37.7	26.0	− 25.5	− 58.6	− 84.1	− 21.3	− 442.0	− 37.7	− 0.015
H_2_O	NH_3_	26.4	40.6	− 29.7	− 59.4	− 89.1	− 22.1	− 453.8	− 32.7	− 0.027
NH_3_	H_2_O	16.7	18.0	− 13.4	− 27.6	− 41.0	− 6.3	− 250.3	− 17.6	− 0.003
NH_3_	NH_3_	18.8	28.5	− 16.3	− 38.5	− 54.8	− 7.5	− 237.3	− 23.9	− 0.013
HF	N_2_	7.1	18.4	− 8.8	− 23.9	− 32.7	− 7.2	− 74.1	− 12.1	− 0.007
HF	F^−^	246.6	64.0	− 285.1	− 269.6	− 554.7	− 244.1	− 751.4	− 182.9	− 0.124

6 $${E}_{\mathrm{def}}^{\Omega }={E}_{\mathrm{intra}}^{\Omega }-{E}_{\mathrm{free}}^{\Omega }$$

Note that a “free atom” (and thus ${E}_{\mathrm{free}}^{\Omega }$) can refer to both a single atom in vacuo as well as to an atom in a free or isolated molecule. Hence, “free” can refer to a group of atoms, forming the molecule that one wants the deformation energy to refer to. Changing the reference energy just alters the zero the energy scale. If one wants to study two interacting molecules, it may make more sense to define each molecule as a quantum fragment. One thus applies an Interacting Quantum Fragments (IQF) analysis, strictly speaking, rather than an IQA analysis.

In analogy with the summation explained above, we can then also sum ${E}_{\mathrm{def}}^{\Omega }$ over the atoms in the proton donor (D) monomer of the hydrogen-bonded complex to obtain ${E}_{\mathrm{def}}^{D}$, and similarly the atoms in the proton acceptor (A) monomer to obtain ${E}_{\mathrm{def}}^{A}$. From Table 1, it is clear that the acceptor molecule is always more deformed (costing more energy) than the donor molecule except for the water dimer and the HF…F^−^ system.

Secondly, we define the traditional electrostatic energy between two atoms *A* and *B* from the Coulomb energy ${V}_{\mathrm{ee},\mathrm{C}}$ (see Eq. (5))

7 $${V}_{elec}^{AB}={V}_{ee,C}^{AB}+{V}_{\mathrm{ne}}^{AB}+{V}_{\mathrm{en}}^{AB}+{V}_{\mathrm{nn}}^{AB}$$

by bringing in the nuclear charge density to balance the purely electronic Coulomb interaction ${(V}_{\mathrm{ee},\mathrm{C}}^{AB}$), where *n* refers to the nucleus of the atom under whose superscript it directly appears. Thirdly, the full interaction energy (electrons and nuclei) between atoms *A* and *B* is defined as

8 $${E}_{\mathrm{int}}^{AB}={V}_{\mathrm{ee}}^{AB}+{V}_{\mathrm{ne}}^{AB}+{V}_{\mathrm{en}}^{AB}+{V}_{\mathrm{nn}}^{AB}={V}_{\mathrm{elec}}^{AB}+{V}_{\mathrm{ee},\mathrm{Xc}}^{AB}$$

which can again readily be generalised to the interaction energy between the donor (D) and acceptor (A) molecule, ${E}_{\mathrm{int}}^{DA}={V}_{elec}^{DA}+{V}_{Xc}^{DA}$, by simple summation of the participating atoms, where DA (a shorthand for D…A) refers to the interaction between D and A. Fourthly, the supermolecular complex’s binding energy *E*_*bind*_ is defined as

9 $${E}_{bind}=\sum_{\Omega }{E}_{def}^{\Omega }+\sum_{{\Omega >\Omega }^{\mathrm{^{\prime}}}}{E}_{\mathrm{int}}^{{\mathrm{\Omega \Omega }}^{\mathrm{^{\prime}}}}$$

which shows that binding is ultimately the result of attractive interatomic interactions partially counteracted by the atoms’ positive deformations. This binding energy is typically associated with the strength of the hydrogen bond. For example, one may read off Table 1 that “the water dimer is held together by a hydrogen bond” of about 21.3 kJ/mol (the typical 5 kcal/mol, familiar to many). Note that each fragment’s (i.e. molecule’s) *E*_*def*_ is obtained by subtracting the total energy of the optimised molecule in vacuo. Group (i.e. fragment or molecule) deformation energies thus include the so-called preparation energy due to the rearrangement undergone by the molecule in transitioning from its in vacuo geometry to the interacting geometry.

At atomic (rather than monomeric) level, it is useful to inspect the electrostatic energy of the hydrogen bond itself, ${V}_{elec}^{HB}$, involving only the hydrogen-bonded hydrogen atom and the base atom it is bonded to (N, O, or F). Table 1 provides this energy, as well as the exchange–correlation counterpart ${V}_{Xc}^{HB}$. Finally, the charge transfer between the donor and acceptor molecule is gauged by summing the net atomic charges of the donor (just by choice), Q^D^. Table 1 shows that electronic charge (expressed in the atomic unit of “electron, e”) always migrates from the acceptor to the donor because all values of Q^D^ are negative.

A final note concerns the relation of IQA to other energy decomposition schemes. The IQA energy terms used in this article are well-defined and consistent within the context of IQA. Already in 2006, work [60] of the Oviedo group made careful comparisons between IQA and SAPT, [61] the paradigm of modern perturbation approaches. Hence, there is a perspective for comparing IQA and non-IQA methods, and IQA is not isolated. However, in order to maximise the extent of comparison, that is, ensuring that energy terms mean the same, physical principles may have to be violated (such as the Pauli principle). The same may be true for published comparisons [60, 62] between IQA on the one hand and KM, [63] NBO, [64] EDA, [65] or NEDA [66] on the other.

### 2.3 Covalency is a sliding scale

**Fig. 3:**
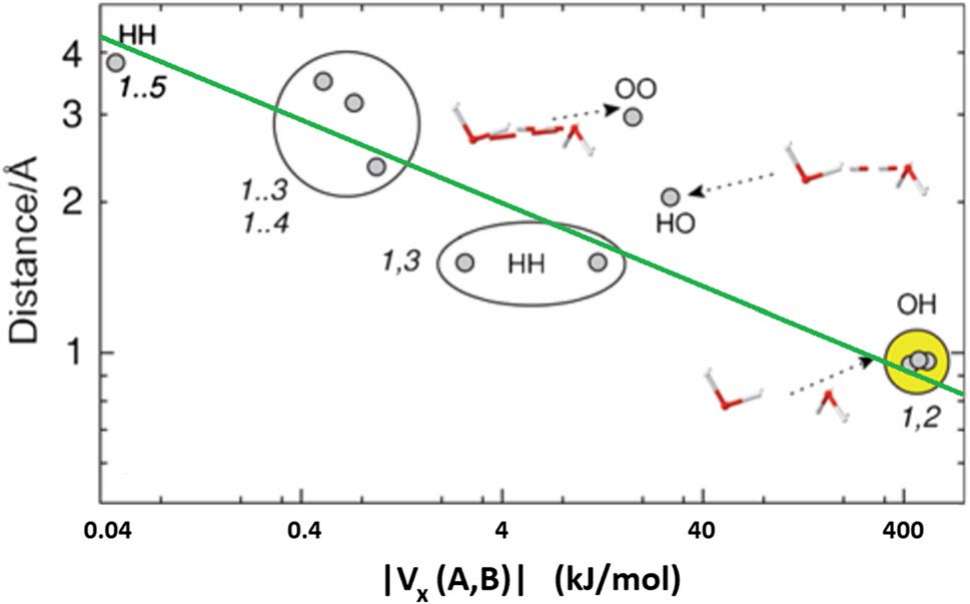
Logarithmic plot linking |V_X_(A,B)| with interatomic distance for the global energy minimum of the water dimer calculated at CAS [5, 6]//6-311G(d,p) level of theory. The green line outlines the broad effect that |V_X_(A,B)| increases with decreasing distance

The interatomic exchange energy, denoted V_X_(A,B), quantifies the covalent character of the interaction between any two atoms *A* and *B* no matter how far apart. The fact that V_X_(A,B) adopts a range of values undermines the traditional binary picture of covalent versus non-covalent interactions. A systematic study [67] of V_X_(A,B) values for dozens of simple but representative compounds proves this point alongside many other interesting points whose discussion is precluded by space restriction. It is informative to plot the relationship between |V_X_(A,B)| and internuclear distance d(A,B), as is done in Fig. 3 for the global minimum of the water dimer.

As anticipated, the molecular Lewis diagram is recuperated from its clear signature in such a plot. Indeed, all molecules clearly show a cluster housing all expected (covalent) bonds and thereby recovering the stripes in the Lewis diagram (or the sticks between the balls in a 3D ball-and-stick model). These so-called 1,2 interactions (originating in force field language) appear as rather isolated clusters of several hundreds of kilojoules per mole. Their island-like nature perhaps instigates the perception of the black-and-white covalent/non-covalent divide. But then the 1,3 interactions (where now two covalent bonds link the two nuclei of interest) can be associated with still handsome values for |V_X_(A,B)|, of the order of 10 kJ mol^−1^. The 1,4 interactions are typically again weaker than the 1,3 interactions, by an order of magnitude or so.

A look at all 1,n interactions (including n > 4) in molecular (rather than metallic-like or strongly conjugated (e.g. polyaromatic)) systems exposes an exponential decay of |V_X_(A,B)| with increasing distance. Certainly one can draw a broad line that connects the various 1,n clusters. However, it turns out that hydrogen bonds appear somewhat set aside from this line. For example, the classical O–H…O hydrogen bond occurring in the global minimum of the water dimer has a |V_X_(A,B)| value that is too high for its internuclear distance according to that overall connecting broad line. This means that the O…H bond is more covalent than expected. Moreover, the O…O interaction is also anomalously strong (i.e. covalent) for its internuclear distance. When taken together, both observations suggest that hydrogen bonding should actually be seen as a three-atom phenomenon, involving the donor atom (D), the acceptor atom (A), and the hydrogen atom. Truly, in a D-H…A system, both |V_X_(D,A)| and |V_X_(H,A)| values are anomalous because they do not appear in the expected place in the V_X_(A,B)- d(A,B) plot.

**Fig. 4:**
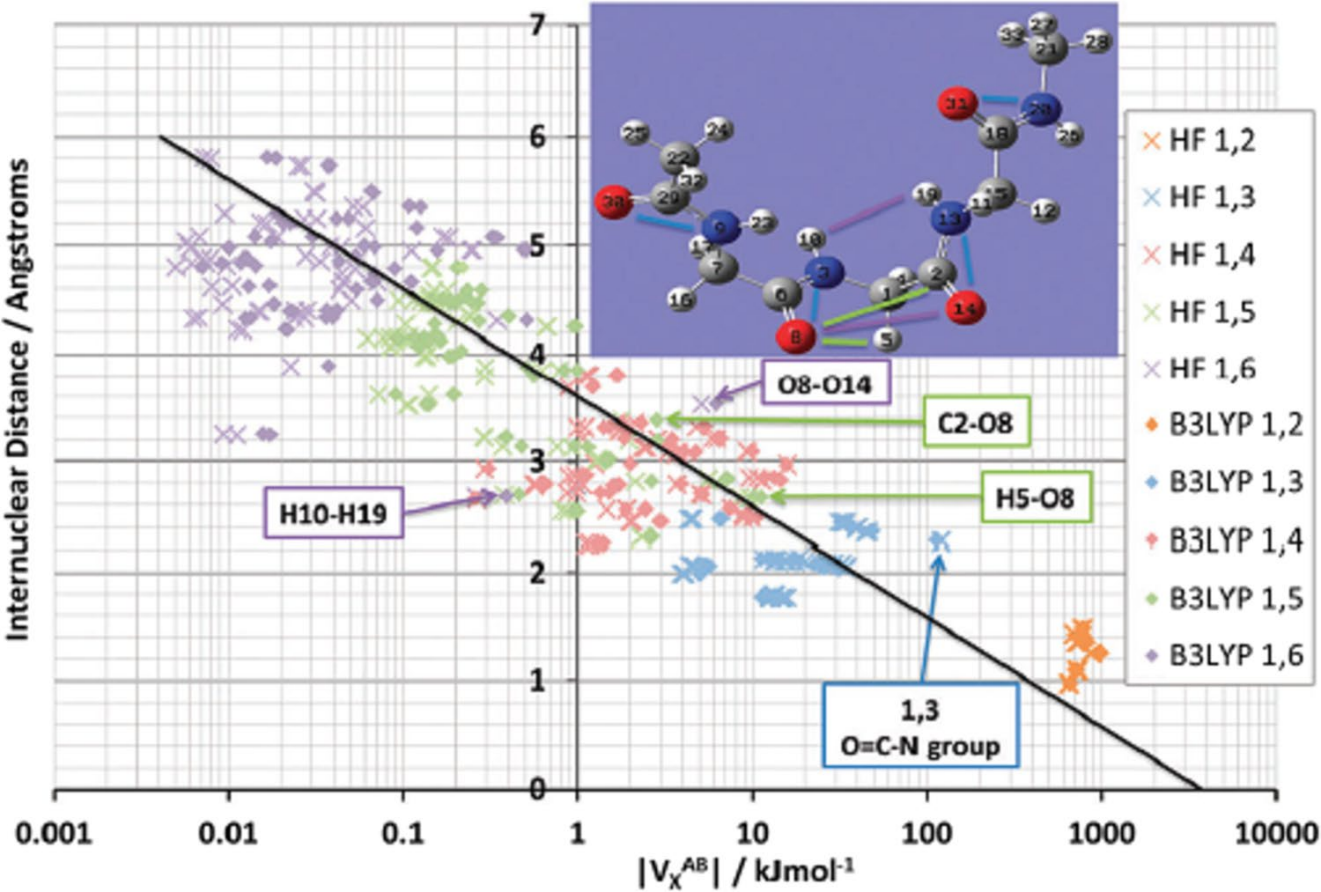
Logarithmic plot of interatomic distance versus |V_X_(A,B)| for Gly-Gly-Gly interactions up to 1,6, calculated at both HF/6–31 + G(d,p) and B3LYP/6–31 + G(d,p) level of theory. Key outliers of V_X_ have been labelled in both the plot and the insert molecular image. The black line shows the overall correlation of the B3LYP energies, with a correlation coefficient *r*. [2] of 0.91

The 529 interatomic interactions of the oligopeptide GlyGlyGly have also been studied [50] by such a plot (up to 1,15 interactions) as shown in Fig. 4. Again the broad line emerges (now in black), connecting very weak interactions (hundredths of kJ mol^−1^) to the covalent bonds of the completely recovered Lewis diagram (hundreds of kJ mol^−1^). Curiously, the |V_X_(N,O)| values arising in the four peptide groups are unexpectedly large. This N…O “through space” contact occurs in the O = C-N group and suggests that the peptidic CN bond is harder to break than expected. As a reason for this observation, one can think of the extra “glue” offered by the N…O interaction stabilising the peptide group.

A second case study in our work [50] focused on alloxan, a heterocyclic planar molecular. The stability of its crystalline form is puzzling because it lacks any hydrogen bonds, which usually fulfill the role of stabiliser. For 40 years, this crystal structure has been regarded as “problematic” until in 2007 Dunitz and Schweizer suggested [68] that it may be explained by important attractive interactions of the type C = O…C = O. Interestingly, strong intermolecular |V_X_(C,O)| values were found (stronger than intra-molecular 1,4 interactions), of the order of 20 kJ mol^−1^ each, which supports their hypothesis.

In summary, the image that QTAIM leaves one within its description of molecules and their aggregates (condensed matter) is one of “bubbles”. These are the space-filling, topological atoms, which initially appear without sticks, that is, if the system if thought of in terms of balls and sticks in the first place. Each bubble interacts with any other and their shapes do not give away which atoms are bonded to which. Bonding patterns, of various strengths, then emerge from the energy term V_X_, which acts as a covalency quantifier. We return to the issue of bonded versus non-bonded interactions in Section 2.5 in connection with force field planning but first we follow through the lack of overlap between topological atoms.

### 2.4 The full consequence of no overlap

The space-filling nature of topological atoms means that there are no gaps between them. This is the correct topological picture, which applies to condensed matter. However, in 1987, Bader et al. [69] defined and calculated atomic volumes, occurring in gas-phase molecules, by considering practical, finite edge to a molecule. This view was based on the concept of collision diameters, which seem to endow molecules with some finite volume, often based on the *ρ* = 0.001 a.u. or 0.002 a.u. constant electron density envelope. Similarly, the traditional (non-topological) picture often shows atoms on different molecules being separated by portions of empty space. For example, the Corey-Pauling-Koltun (CPK) picture portrays molecules as having an abrupt edge, presenting them as macroscopic objects that can literally be grabbed.

The consequence of the space-filling nature of condensed matter (e.g. a ligand inside a protein pocket) is that each point in space must belong to an atom; there is no “empty” unassigned space. The full impact of this fact must still be worked out in connection with protein–ligand docking [70]. Surely, the CPK picture may just be for visual convenience mainly, while traditional quantum descriptions think of molecules as never ending (overlapping) clouds. These two pictures clash and both interpretations disturb energy book keeping, actually. Electrostatic energy, for example, is mathematically connected to electron density (see the first term of Eq. (5)). Thus, if some “empty space” electron density does not belong to an atom then the associated electrostatic energy does not either. This means that some energy will be unaccounted for. Equally, if some electron density simultaneously belongs to two atoms then energy will be double-counted. In summary, space-filling atoms present a clean and minimal picture, certainly when constructing a force field because all energies must be associated with nuclei.

**Fig. 5:**
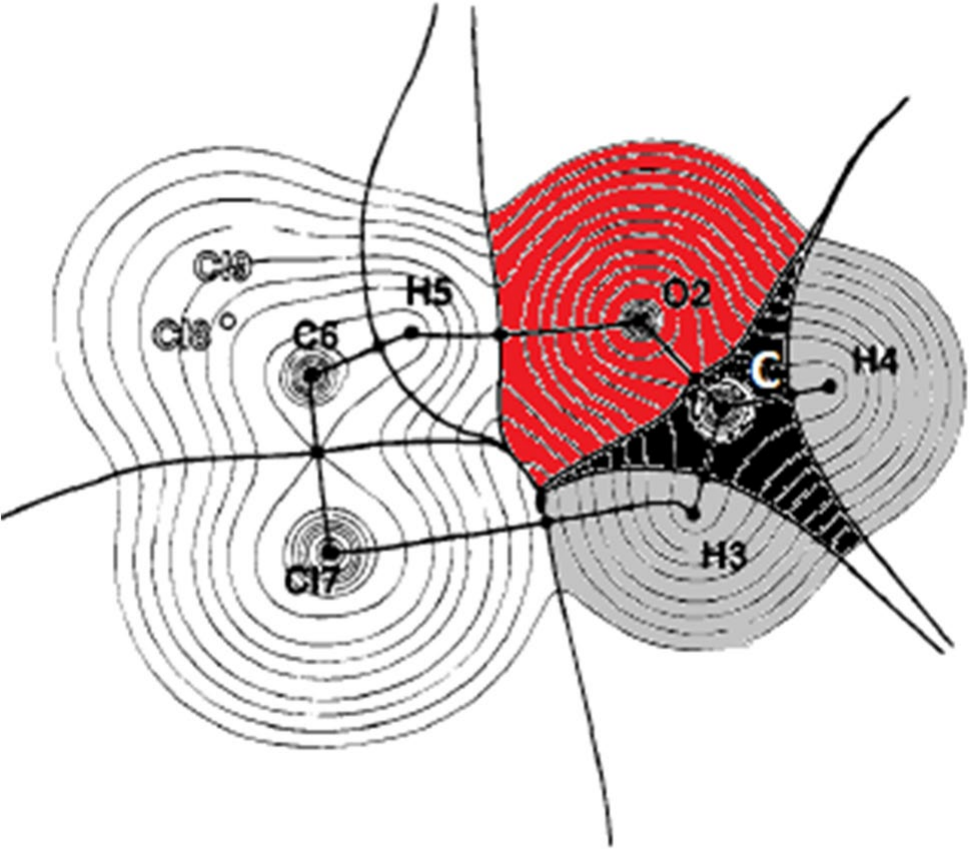
An example of the non-overlapping nature of topological atoms as they occur in a methanal…chloroform van der Waals complex. The atoms in methanal are coloured for clarity

Figure 5 shows an example of the space-filling character of topological atoms in an intermolecular context. It is clear that the two molecules forming a van der Waals complex do not overlap; instead, they indent each other. If the complex were strongly compressed, then the molecular distortion would increase but the respective atoms would remain well-defined, and so would their properties.

This fully non-overlapping picture is the purest (in the world of QCT) that one can work with but it can be diluted. In that case, a “halfway house” compromise appears in which the molecules themselves consist of non-overlapping atoms but the molecules are allowed to overlap each other. This route was followed in a study [71] of the convergence behaviour of the electrostatic interaction, allowing for two separate and overlapping monomeric wavefunctions. It should be mentioned that this convergence behaviour was also investigated [42] for atoms appearing in supermolecular wavefunctions, which corresponds to the proper topological view of non-overlapping monomers. Further reflection on the nature of overlapping objects has been published in the context of clouds [72] in the sky and even colliding galaxies [73].

“Monomeric simulations” can still be carried out (for example refs [74–76]) as a first approximation by allowing the “topological sacrilege” of overlapping molecules. The force field FFLUX, [77] which is still under construction because of its novel architecture and its *tabula rasa* origin, embraces the idea of non-overlapping molecules. We strive to work out the full impact of that idea once transferability has been incorporated into FFLUX. FFLUX uses the machine learning method kriging [78] to map the atomic energies and multipole moments (output) of a given atom onto the coordinates of the atoms surrounding it (input). How to do this for the atoms in a central water molecule inside a water decamer, for example, has been shown before [79].

### 2.5 Bonded versus non-bonded interactions

The construction of classical force fields is strongly influenced by the binary divide between bonded (i.e. covalent) and non-bonded (non-covalent) interactions. The bonded interactions are typically of the types 1,2; 1,3; and 1,4. These interactions are solely modelled by bond-stretching potentials (e.g. harmonic, cubic, or even Morse-like), valence potentials, and torsion potentials. The non-bonded interactions (1,n; n > 4) are then suddenly modelled by an electrostatic potential. An honest and innocuous question is why bonded atoms do not interact electrostatically too? Of course, physically they do. Undeniably, within the IQA *ansatz*, the quantity ${V}_{\mathrm{ee},\mathrm{C}}^{AB}$ appearing in Eq. (5) is not restricted to *A…B* interactions of the type 1,n > 4. So classical and many next-generation force fields have a non-physical dichotomy at the heart of their design. Perhaps this dichotomy causes the documented ambiguities in energy representation at the level of 1,4 interactions, which are at the border between bonded and non-bonded interactions. Similarly, hydrogen bonds have witnessed a checkered history in the modelling of their energies, with dedicated potentials being added and then eliminated again during the typically protracted chronology of force field development.

A variant of the innocuous question above is why the dispersion interaction does not operate between bonded atoms either in the familiar potentials of force fields. For sure, the dispersion part of the Lennard–Jones potential only seems to act between non-bonded atoms. Looking at Eq. (5) shows that now the atomic electron correlation, denoted ${V}_{\mathrm{ee},\mathrm{c}}^{AB}$, is again not restricted in terms of 1,n types. Indeed, dispersion should be covered by the dynamic correlation behind ${V}_{\mathrm{ee},\mathrm{c}}^{AB}$. Such correlation energies can be routinely calculated at the Møller-Plesset level, initially for very small systems [80] and then upscaled [81] to MP2 correlation energies for bonded and non-bonded interactions in a deprotonated and hydrogen-bonded glycine…water complex, for example. FFLUX is planned to also incorporate this type of energy contribution and thereby break the artificial bonded/non-bonded barrier. Although a proof-of-concept to machine learn ${V}_{\mathrm{ee},\mathrm{c}}^{AB}$ was recently reached, [82] the enormous size of the two-particle density matrix causes the concomitant computation to be very slow too. Work to tackle this challenge is in progress in our lab.

### 2.6 No perturbation theory

A long time ago, it was decided that FFLUX should not to be developed within the context of long-range Rayleigh-Schrödinger perturbation theory [83].  Intermolecular forces at long range are traditionally treated according to this formalism, which operates when the overlap between interacting moieties is small (although textbooks do not seem to quantify “small”). The very idea of perturbation theory has a stronger imprint on classical and next-generation force fields (listed above) than expected at first glance. This imprint consists of the rigid-body nature of the molecule being perturbed. Force fields that incorporate advanced treatments such as distributed multipole moments or polarisabilities were originally limited to handling rigid fragments. This imprint also has an impact on polymorphism prediction, [84] for example, when using force fields. However, the introduction of machine learning into the world of topological atoms has freed FFLUX from the rigid-body shackles. It is possible [85] to carry out simulations with flexible water molecules whose (high-rank) atomic multipole moments vary with the water molecules’ geometries.

Let us return to “monomeric” molecular dynamics simulation. Here, one allows single molecules to interact via their gas-phase (isolated) wavefunctions. In principle, polarisability can be added [86] to the potential governing the simulation. Instead of introducing a single polarisability tensor for the whole molecule having, it is better to work with atomically distributed polarisabilities. However, in our group (so not in general), this route was abandoned early on. This local decision was taken in spite of the fact that the topological partitioning generates polarisabilities of excellent stability [87] with respect to basis set variation. Note that some non-topological (atomically distributed) polarisabilities [88] suffer from instability but ISA-Pol is basis set stable. What is then the ultimate strategy (“Plan A”) for FFLUX?

First, Plan B is discussed briefly because it is easier and has already been realised. We are currently running monomeric simulations on liquid water with high-rank multipolar electrostatics implemented by Smooth Particle Mesh Ewald (SPME) summation. A given water, call it central, interacts with the electric field generated by the surrounding waters. This interaction causes electrostatic forces on the nuclei. In turn, these forces cause a geometry change inside the (flexible) water molecule. This change leads to a new intra-molecular energy, which is predicted by machine learnt models, one prediction for each (new) atomic energy. The machine learning only needs the input of that new geometry to make its predictions. Secondly, concomitant models predict the new multipole moments for that geometry. These new moments create a new electric field such that another iteration of energy and geometry adjustment can happen. The whole process is a “negotiation” of intra- and intermolecular energy, which makes the geometry of each participating water molecule fluctuate.

Note that any polarisation effects are based on the change of electron density within a single gas-phase molecule. Unfortunately, these effects are small compared to the more realistic situation where a change in electron density (and thus multipole moments) is obtained from a water wavefunction calculated in an electric field. One could go down this route (and call it Plan B) but it is tempting to take up the challenge of Plan A right away. This plan involves training the models for pieces of matter larger than a single molecule, for example, a water dimer or trimer. We call this “oligomeric modelling”, which will automatically take into account how the electron density of an atom inside an oligomer changes. This change will cover all polarisation effects, including that due to partial covalency in hydrogen bonds and to many-body influence. In summary, FFLUX treats polarisation, not by focusing on the *process* of polarisation (i.e. polarisability) but on the *result* of this process (i.e. the final multipole moments including charge (or 0^th^ moment)).

According to the polarisation approximation, [89] the energy of two interacting ground-state molecules *A* and *B* consists, up to second order, of the sum of (i) the individual molecules’ energy (0^th^ order), (ii) the purely electrostatic energy between *A* and *B* (1^st^ order), and (iii) the induction energy of *A* and of *B*, and the dispersion energy between *A* and *B* (2^nd^ order). These energy contributions are well-defined *within this particular formalism*, and can be calculated accordingly. However, how robust is the definition of dispersion outside the polarisation approximation?

Imagine a practical case where the formal distinction between intra- and intermolecular interaction is blurred. Take a chainlike molecule and curl it such that its two endpoints are facing each other over a short distance. The atoms of these endpoints interact as if they were part of two different molecules. In other words, if the chain were not shown in full, then one would not know that these terminal atoms are actually part of the same molecule, that is, a curled chain. The formal difficulty is that the unperturbed Hamiltonian cannot be written as a sum of two Hamiltonians, one for each *isolated* fragment *A* or *B*, because there are no two fragments. Instead, there is a single molecule. Thus, one may conclude that the concept of dispersion mathematically dissolves because of the framework of perturbation theory. Yet, there must be dynamical correlation between these two end-of-chain atoms. The topological interatomic correlation energy, $V_{{\text{ee,c}}}^{{}}$, which exists independently of perturbation theory, will pick up this phenomenon. In fairness to symmetry-adapted perturbation theories such as SAPT, [61] however, it is finally possible to formulate an atomically decomposed version called A-SAPT [90]. A melee of partitioning methods is invoked for the different types of energy contributions, running counter to the minimal and streamlined philosophy behind IQA. Alas, A-SAPT often has difficulty producing chemically useful partitions of the electrostatic energy, due to the buildup of oscillating partial charges on adjacent functional groups. This is why, immediately after the presentation of A-SAPT, F-SAPT was proposed, [91] the functional-group SAPT partitioning. F-SAPT could be used to solve the problem of the curling chainlike molecule mentioned above, as well as from an incremental fragmentation method [92]. Note, however, that IQA does not need special constructions to be able to calculate any interaction energies between any two atoms, wherever they occur, within the same molecule or not. The “curly chain molecule” situation is not a problem for this method, atoms being atoms, wherever they are; the wavefunction that provides the atomic electron densities can be any justifiable size, whether a single molecule or an assembly thereof.

A second problem with perturbation theory is that, at short-range, Rayleigh-Schrödinger perturbation theory actually breaks down because there is no unique definition of the order of a term in the perturbation expansion. However, exchange perturbation theories have been proposed, of two main types: symmetric methods (e.g. Stone-Hayes [93]) or symmetry-adapted theories (e.g. SAPT [61]). Then again, however, short-range perturbation theory is computationally expensive because one needs to take into account the other molecules that a given molecule interacts with. This is still true for methods such as SAPT(DFT), which are competitive in terms of accuracy and computational expense. In contrast, no knowledge of surrounding molecules required for long-range perturbation theory. In other words, one can calculate the polarisability of a molecule without having to know which molecule causes the polarisation.

Based on the considerations above, it makes sense to develop FFLUX using supermolecular wavefunctions. The latter are free of any molecular imprint. At a deep level, FFLUX’s architecture does not differentiate between intra- and intermolecular interactions: an atom is an atom wherever it is. Put differently, the electron density of the overall system partitions itself, according to QCT, by following the (nuclear) attractors rather than by where the molecules stop or start. The machine learning is well suited to be trained on atoms that that are sufficiently embedded in a relevant piece of matter.

### 2.7 No penetration, no damping functions

A damping function is a mathematical function that prevents an energy from becoming unreasonably large (and even infinite) at short range. A damping function can occur within the context of electrostatic energy, induction energy, or dispersion energy (e.g. reference [94]). The reason for damping functions can be traced back to the so-called penetration effect, which in turn follows from the (conceptual) picture of electron clouds extending infinitely far and thus being able to substantially overlap with each other. However, if electron clouds do not overlap, as in the topological approach, then no penetration effect emerges. Both the penetration energy and damping function arise from the fact that the point at which we want to know the electrostatic potential resides inside the electronic charge density that generates this potential. However, with a topological atom, it is possible to take a point that is rigorously outside the atom (i.e. the finite object that generates the potential) and calculate [95] the electrostatic potential at that point.

### 2.8 Point-charges for electrostatics versus monopole moments as a measure for charge transfer

In research circles preoccupied with applied biomolecular simulation, the point-charge is still the standard way of handling electrostatic interaction. While more and more ambitious biomolecular problems are tackled at accelerated pace, it is a “suppressed truth” that the results ultimately depend on the quality of the potentials used. The electrostatic component of these potentials is crucial in the polar and charged systems that are biosystems. The examples of non-covalent interactions mentioned in the “Introduction” cannot be treated correctly if the electrostatics are faulty. And they are faulty at short and medium range if an atom’s charge density is represented by only one point-charge.

While several research groups (e.g. references [84, 96–104] amongst others) continue to improve potentials, the essentially stagnant architecture of classical force fields means that ever growing computer power will yield the wrong answer faster, frankly. A recent and dramatic example [105] of the lack of reliability is the comparison of ensembles of intrinsically disordered proteins, generated by eight all-atom empirical force fields, with primary small-angle X-ray scattering and NMR data. Ensembles obtained with different force fields exhibit marked differences in chain dimensions, hydrogen bonding, and secondary structure content. These differences turned out to be unexpectedly large: changing the force field is found to have a stronger effect on secondary structure content than changing the entire peptide sequence! All this vindicates a fresh start in force field design and FFLUX is such an attempt, commenced several years ago.

A second, more modest but still poignant, case study [106] is that on the paradigm molecule trialanine. This thorough computational and experimental study ran 20 ns simulations with six different force fields [Amber (parm94, parm96), GROMOS (43A1, 45A3), CHARMM (1998) and OPLS (all atom, 1996)]. Their conclusion was disappointing: “*…lifetimes of the conformational states differ by more than an order of magnitude, depending on which model*.” Indeed, even the minor modification between “parm94” and “parm96” significantly changed the population ratio of the conformational states.

There is considerable evidence [107] that multipolar electrostatics overcome the limitations of the ubiquitous point-charge approach. In particular, a model of one point-charge for each atom fails to capture the anisotropic nature of electronic features such as lone pairs or π-systems. However, high-rank electrostatic terms (involving multipole moments) naturally recover these important electronic features. One extremity is to add (point) multipole moments centred on the nucleus. The other extremity is to surrender to the obsession of point-charges and add more point-charges per nucleus, away from the nuclear position. This route was followed by the TIPnP family of water potentials, for example. However, a third option, of searching for the elusive point-charge that will magically turn out to be correct at short range, is pointless. No work seems to have been done [108] on comparing the computational cost of using multipole moments versus an equivalent number of point-charges yielding the same accuracy.

A final comment relates to an unfair interpretation [55] of the performance of a point-charge in reproducing the molecular dipole moment or the electrostatic potential. We briefly discuss both, in turn. Claiming that QTAIM charges do not reproduce the molecular dipole moment is misleading because the latter is made up of two contributions, often similar in magnitude. One contribution is the typical one, due to the point-charges. This interatomic charge transfer component is the only contribution considered in naïve and incomplete accounts. The other is the intra-atomic (dipolar) polarisation contribution, which should not be ignored. If one omits this contribution, then one cannot explain the nearly vanishing dipole moment of carbon monoxide, for example. QTAIM clearly explains what happens in CO, and does so with its typically large values for the monopole moments (i.e. charges) but which are consistent with the electronegativity difference between carbon and oxygen. When it comes to the electrostatic potential, a QTAIM charge does not claim to be able to reproduce it well. The argument is that it does not have to do so: an atomic charge is a measure or product of charge transfer, no more and no less. If an electrostatic potential [95, 109] or interaction [71] is to be modelled exactly then atomic multipole moments need to be invoked. The latter are necessary to represent the details of an atomic electron density beyond the first “summary” offered by a point-charge.

### 2.9 Multipolar electrostatics and convergence

We have thoroughly researched [42, 71, 95, 109–116] topological multipolar electrostatics, especially the convergence behaviour of the multipolar expansion. At very long range, the interatomic electrostatic interaction ${V}_{elec}^{AB}$ becomes identical to the usual *q*_*A*_*q*_*B *_*/r*_*AB*_ expression where *q* is a net atomic charge (e.g. − 1.1e for oxygen in water). At closer range, expressions for charge-dipole, dipole–dipole, and dipole-quadrupole are necessary to approximate ${V}_{elec}^{AB}$ better.

**Fig. 6:**
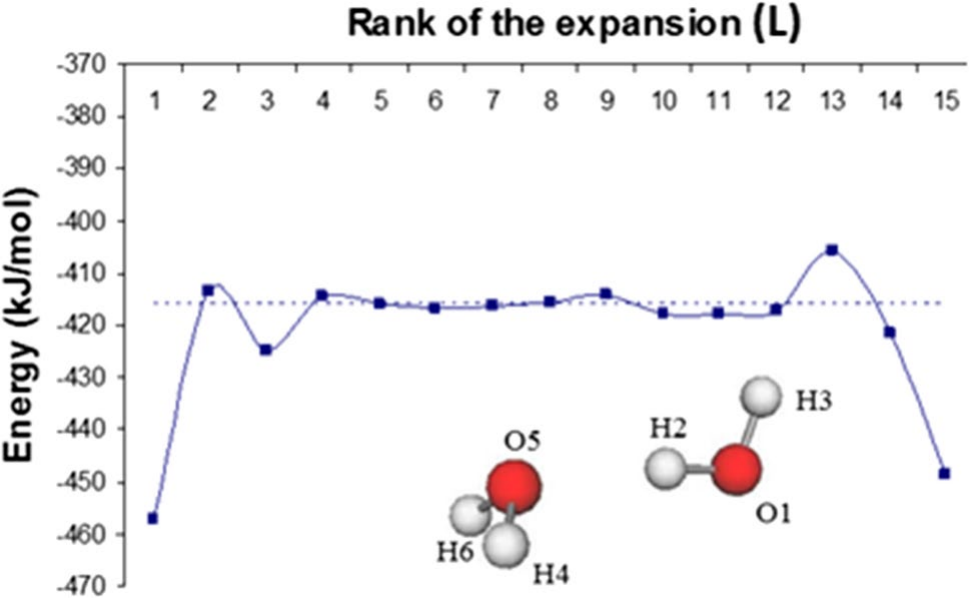
The exact electrostatic interaction energy ${V}_{elec}^{AB}$ between two (topological) atoms (dashed horizontal line) compared with the energy obtained by multipolar expansion according to interaction rank *L* for the hydrogen-bonded atoms (O_5_ and H_2_) in the global minimum of the water dimer [114].

Figure 6 illustrates convergence (or lack thereof) for the case of the electrostatic interaction between the two hydrogen-bonded atoms (O…H) in the global minimum of the water dimer. The interaction rank *L* of the multipolar expansion is defined as

10 $$L={\ell}_{A}+{\ell}_{B}+1$$

where $\ell_{\Omega }$ is the rank of a multipole moment centred on atom Ω. For example, $\ell=2$ for a quadrupole moment, which has 5 (not 6) components in the spherical tensor formalism that we use (instead of the Cartesian one, which contains redundancies).

The energy profile in Fig. 6 shows that the point-charge representation (L = 1) is a poor approximation to the exact electrostatic interaction, ${V}_{elec}^{AB}$, obtained without expansion. The situation improves dramatically at L = 2, with the addition of a dipole moment on oxygen and on hydrogen. Worse agreement then appears at L = 3 but at L = 5 the convergence is essentially exact. This stays so as *L* increases until divergence sets in after L = 13. This whole energy profile is a case of pseudo-convergence because of the convergence breakdown at very high L. However, for all practical purposes, an essentially exact ${V}_{elec}^{AB}$ value can be obtained from the multipolar expansion for a stable plateau between L = 4 and L = 12. Finally, real (full) convergence can be realised while divergence never occurs, no matter how high the interaction rank L. We note that this formal (exact) convergence is elusive if the respective atoms are infinite in size, which is the case for DMA. It would be nice if this topological convergence work of this section were finally reported in a classic and otherwise valuable reference work on intermolecular forces, [83] the second edition of which unfortunately still mentions the same erroneous statement on the poor convergence of topological atoms as in the first edition.

Next, a systematic study [117] on the small protein crambin provides a wealth of information on universal convergence behaviour between the most five common elements of Life (C, H, N, O and S). One of five key questions answered in that study asks: *For a given convergence energy error, ΔE, and a given pair of atoms A and B, how does the interaction rank L at which the energy has converged, change with increasing internuclear distance R?* Essentially this question reduces to when a multipolar expansion can be truncated. This is actually a five-dimensional function, involving the quantities *R*, *L*, and Δ*E*, and the qualifiers* A* and *B.* It is abundantly clear that short-range electrostatics cannot be achieved by multipole moments. Instead, exact electrostatics is achieved via six-dimensional integration over two interacting atoms. However, accurate electrostatic descriptions (within 0.1 kJ mol^−1^ of the exact answer) can be obtained, for example for O…H interactions, if the charges are far apart. An internuclear distance of around 25 Å would be safe to cover all geometries and particular atoms. However, a distance of around 8–9 Å already suffices for some favourable interactions. An example of good news in this context is that all C…C interactions are within 0.1 kJ mol^−1^ of the exact answer already for L = 1 (dipole–dipole), and already around 7 Å.

Finally, molecular dynamics simulations on water clusters (25–216 molecules) with a machine-learnt quantum topological monomeric water potential should be mentioned [118]. Both the intramolecular energy and the atomic multipole moments were trained by Gaussian process regression (aka kriging). This is the first time that multipolar electrostatics “negotiated” its intermolecular energies with the monomers’ intramolecular energy. It turned out that while incorporating charge-dipole interactions into the description of the electrostatics resulted in only minor differences, the incorporation of charge-quadrupole, dipole–dipole, and quadrupole-charge interactions resulted in significant changes to the intermolecular structuring of the water molecules.

### 2.10 Polarisation

Typically, anisotropic polarisabilities handle the complexity of the electron density responding differently depending on direction, and capture the subtleties for all multipole moments. Long-range perturbation theory is the traditional framework in which this approach is formulated. However, one could argue that long-range perturbation theory has two conceptual drawbacks when it operates at too short a range (and SAPT is not invoked): (i) it handles charge transfer in a “bolt on” manner, and (ii) it causes the polarisation catastrophe. A further drawback is more practical in nature: a polarisation scheme governed by a polarisability tensor introduces computational overhead during a molecular simulation. This is so because, for each number of time steps, the new multipole moments resulting from each polarisation process must be computed “on-the-fly” in an iterative manner, until self-consistency is reached.

In order to tackle these drawbacks, we proposed [119] an approach that does not focus on the polarisability itself but on the effect that it has, after the polarisation process has done its work, as it were. The machine-learning-based force field FFLUX embraces this idea and thereby predicts the new multipole moments that an atom must adopt when in the presence of a new atomic environment. A machine learning method is basically a mapping between inputs and outputs, and needs to be trained to achieve a successful mapping. This success is measured by an objective function, which is essentially the difference between an original output and a predicted output. This difference (i.e. prediction error) should be minimised and this is best done by comparing the predictions against an external test set, i.e. data points that do not appear in the training set. FFLUX took up a method called kriging or Gaussian processes in order to predict the effect of polarisation. However, we originally used [120] an artificial neural network but this methodology was abandoned [121] in favour of kriging because the latter is more accurate albeit more CPU intensive. An important advantage of kriging is that it handles a high-dimensional input space better than neural networks.

It is important to realise that all multipole moments (including monopole and dipole moment) are treated in the same way. Essentially, charge transfer is the effect of “monopolar polarisation”. Thus, charge transfer is not in need of special handling but is just another special case of multipolar polarisation. Hence, unlike in perturbation theory, FFLUX handles charge transfer and multipolar polarisation in a unified [122] and streamlined manner. Having a robust way of defining atomic charges is pivotal to make this uniform treatment a reality but, earlier on, QTAIM (which underpins FFLUX) has been argued to do so.

### 2.11 The bond critical point (BCP)

As discussed in Section 2.2, QTAIM started as an energy partitioning method in 1972. While the concepts of atomic basin and interatomic surface had crystallised first, the concept of a bond path appeared only 5 years later. A bond path was introduced [123] as the two gradient paths that originate at the “internuclear saddle point” and terminate at each of the thus connected nuclei. The name bond critical point (BCP) then appeared [124] another 2 years later. A critical point is a point in space where the gradient of the electron density vanishes. Inspecting the eigenvalue spectrum of the Hessian at a critical point gives four types of critical point: a minimum, a maximum, and two types of saddle point [5, 7, 8]. The BCP is a minimum in the direction of the bond path and a maximum in the two directions orthogonal to this path, at the critical point. It is important to realise two matters: (i) the BCP point was coined by observation; that is, it appears between nuclei that everyone agrees on are bonded, by a standard covalent bond, and (ii) there is no connection between the BCP and the energetics of the virial partitioning proposed earlier, which is at the heart of QTAIM.

The four critical points can be characterised by their so-called rank and signature. This is a purely mathematical characterisation without any reference to the chemical meaning behind a critical point. The reason why this comment is important, especially for the bond critical point, will be clear at the end of this section. The critical points can be attributed names such as ring critical point (one type of saddle) or cage critical point (a minimum). Importantly, neither name carries any chemical content: they only describe the geometrical (or even topological) relationship that the critical point has with its environment. Only the bond critical point is “loaded” with chemical meaning because its name goes beyond pure geometry, topology or its mathematical name of (3, − 1). While the name BCP is fit for purpose for standard covalent bonds, it will soon be clear that this is not the case for non-covalent bonds. The latter are those interactions for which the community has an increasing and urgent need to know if a BCP can help in proving the localisation of non-covalent interactions and the measurement of their strength. However, BCP patterns helped [125] defining the molecular structures of non-trivial covalent bonds in *closo-*, *nido-*, and *arachno*-boranes.

Already in the 1980s, the Bader group knew [126] that an external condition was necessary to make a BCP an indicator of a bond. More precisely, a bond path only indicates a bond when the forces on all nuclei in the system vanish. Otherwise, the “bond path” is actually a so-called atomic interaction line. The external condition of vanishing forces solves a problem occurring with Hartree–Fock wavefunctions of noble gas dimers. For example, for He_2_, there is no energy minimum at any finite internuclear distance for a Hartree–Fock wavefunction. Thus, the forces on the nuclei never vanish and the atomic interaction line never becomes a bond path. Thus, there is no bond between the two He atoms, which is consistent with the Hartree–Fock energy profile. As such, the interpretation remains consistent but at the expense of an external condition. However, very recently it has been suggested to improve the nomenclature for the general (non-covalent) case, and call [127] a BCP a line critical point. [128] This critical point’s name is then on a par with the other critical points and devoid of any judgement on what it means chemically. The same can be done for the bond path, which then is better called a line path.

Similarly to standard covalent bonds, QTAIM provided, [129] already back in 1988, a non-controversial topological picture of standard hydrogen bonds. These occurred in a dozen or so simple van der Waals complexes of the type base…HF. Four of these systems were revisited [60] much later (alongside 5 more elementary complexes including F–H-F^−^) with more sophisticated topological tools such as IQA. Also, in the late 1990s a successful and widely used relationship was proposed, [130] based on 83 X–H…O (X = C, N, or O) hydrogen bonds, experimentally observed by accurate X-ray diffraction. This simple relationship links the hydrogen bond dissociation energy (calculated at HF level) with the potential energy density (V) evaluated at the hydrogen-bond BCP. So, in partial summary, all was still well up to that point, when staying with standard covalent and non-covalent interactions. However, from the early 1990s onwards, work started to appear that called into question the presence of a BCP as a signature of an attractive interaction that one calls a bond. A gaggle of seven papers, ending with one on torsional motion in biphenyl, [131] feverishly discussed systems (ranging from C(NO_2_)_3_^−^, over kekulene and biphenyl, to push–pull hexasubstituted ethanes) that had some unexpected BCPs. The authors changed their mind about whether the BPCs that they saw, represented bonds or steric interactions. The history of this debate can be found in Section 4.3 of reference [73] alongside Bader’s utter rejection [132] of the notion of steric repulsion expressed as “interaction lines” (i.e. the collection of gradient paths springing from the “BCP”). This 56-page essay, [73] about half of which is on bonding, reflects on these matters in a way that is as relevant today as it was 15 years ago, including fresh ideas that have still not been explored further. However, in 2019, promising progress was made on the case of biphenyl, [133] a system with its own controversial history spanning almost three decades, which may well have been closed by this recent work.

It is worth spending a paragraph on the important case of biphenyl because we consider it solved [133] by the so-called Relative Energy Gradient (REG) method, [134] which is briefly explained in the next section. The controversy concerns the BCP that appears between two *ortho*-hydrogens (each hydrogen belonging to a different phenyl ring) as the central torsion angle is rotated from its value of ~ 45° (at equilibrium) to a value below 28°, en route to the planar conformation. This BCP was originally interpreted [131] as a signature of the repulsive interaction between *ortho*-hydrogens, a steric clash to which the origin of the torsional rotation was ascribed. The REG analysis disproved this interpretation. REG looks at all possible intra- and interatomic energies (of all IQA types) and does not project a chemical view onto the individual energy profile, even if following chemical intuition. Instead, REG finds out, in a minimal, unbiased, and mathematical way, which atomic energy profile acts most like (or unlike) the total energy profile. The REG study concluded [133] that the planar energy barrier is caused by the inner destabilisation of the two *ortho*-hydrogens, which is equivalent to the textbook steric clash. However, this destabilisation is partially counteracted by the formation of a weak covalent bond between the *ortho*-hydrogens. When energy types are summed, this partial cancellation diminishes the role of the *ortho*-hydrogens. As a consequence, the REG analysis actually identifies the energy behaviour of the C_ortho_ atoms as the cause of the planar barrier. This means that the role of the BCP as a signature of an attractive interaction is preserved. This conclusion also increases confidence in the status [135] of hydrogen-hydrogen [136] bond paths as markers of stabilising interactions in molecules and crystals, as opposed to nonbonded steric repulsions. But then again, in 2016, an extensive study [137] on critical points (and molecular graphs) of promolecular densities of unsubstituted hydrocarbons was published. It showed that the promolecular densities yield the same number and types of critical points for 90% of the hydrocarbons as the real molecular electron densities. The conclusion stated that “*the topology of the electron density is not dictated by chemical bonds or strong interactions and deformations induced by the interactions of atoms in molecules have a quite marginal role, virtually null, in shaping the general traits of the topology of molecular electron densities of the studied hydrocarbons, whereas the key factor is the underlying atomic densities*.” If IQA’s interatomic exchange energies were introduced to vindicate the covalent nature of the allegedly bonded atoms at stake, then promolecular criticism would collapse. Indeed, all energies would vanish for such promolecular wavefunctions and the BCPs would be exposed as false signatures.

Further damage was done to the elegant but increasingly frail idea that BCPs could be used as simple, and indeed computable, indicators of bonding. A few, but then already sufficient, recent computational experiments [138] showed the horrors that can be inflicted on BCP interpretation, at least to those who wish BCPs to succeed in their originally intended remit. For example, a uniform external field polarising a high-level-of-theory electron density of H^−^ creates a BCP, and so does the positioning of a proton in its vicinity.

A natural question that arises from all this alarming news is: what is the link between the appearance of a BCP and an IQA energy balance? After all, the latter can be trusted as an information source to decide which atoms considerably attract each other as judged by their ${V}_{x}^{AB}$ values. A remarkable answer came from a paper published [139] in 2007, which looked at the classical water formation from O(^1^D) and a [1]${\Sigma }_{\mathrm{g}}^{+}$ H_2_ molecule. This system was already studied [124] in 1979 for the topological change that it displayed as the oxygen approaches the hydrogen molecule along its perpendicular symmetry axis. The 2007 work monitored the exchange–correlation energy (${V}_{xc}^{AB}$, which is dominated by ${V}_{x}^{AB}$) during the oxygen’s approach. During this process, the H–H bond weakens while the O–H interaction strengthens (covalently). The key point is that these two interactions compete. It was observed that OH defeats HH (i.e. $|{V}_{xc}^{OH}|\ge |{V}_{xc}^{HH}|$) very closely to the point where HH’s BCP disappears at roughly the same time that the BCP of the O–H bond in water appears. This story is slightly simplified because there is a fleeting topological ring involved but the astonishing fact remains that an interpretational connection between energy and topology was established for the first time. A second such classical case is that of the HCN isomerisation to CNH, which shows the same connection.

About 6 years later, Tognetti and Joubert introduced [140] a simple measure (called β) to quantify the competition between atomic interactions. For 36 systems of the type O…X (X = O, S, or halogen), they found that the value of β, which is a ${V}_{x}^{AB}$ ratio between primary and secondary interactions, determines where the BCP will appear. Again about 6 years later, Jabłoński refuted this approach in a paper [141] with an unusually aggressive title. Incidentally, this paper suitably introduces the trials and tribulations of the interpretation of BCPs extensively but could benefit from a couple of additions. Instead of repeating this history here, it is more fruitful to mention these two additions. One addition is a popular method, [142] unhelpfully^1^ called non-covalent interactions (NCI), and based on the simple observation and assumption that the combination of a vanishing reduced density gradient (a fundamental dimensional quantity in DFT) and low electron density identifies a non-covalent interaction. When this gradient vanishes, one actually ends up with a critical point in the electron density. So, practically, this method actually boils down to spotting BCPs but with the get-out clause of finding a “near critical point”. Indeed, the method highlights a zone around an elusive BCP by plotting some low-value iso-surface of the reduced density gradient. Of course this procedure gets around the thorny problem of a BCP appearing or disappearing upon a small variation in the system’s geometry. Because of the relaxed “critical point” allocation of NCI, it can claim [143] that QTAIM’s criteria are too stringent and they can thus miss an intramolecular BCP in 1,*n*-alkanediols, for example; miss it exactly there where a red-shifted OH-stretching vibrational mode says there should be a hydrogen bond. In summary, as a result of the contour surface this method is, strictly speaking, not part of the topological approach (although inspired by it) and will not be discussed further. The second addition is unique work [144] on BCP distributions collected from a molecular dynamics simulation of an ethanol…water mixture. This dynamic study of BCPs looked beyond static BCP patterns thereby eliminating the ephemeral nature of the BCP in terms of its presence sensitively depending on the exact nuclear geometry. It was found that the more localised such a dynamical BCP distribution, the higher the average electron density at its BCPs. Furthermore, the hydrogen atoms of water strongly preferred to form H…H interactions with ethanol’s alkyl hydrogen atoms over its hydroxyl hydrogen.

### 2.12 Relative Energy Gradient (REG) method

Before explaining REG it is useful to consider the general context that motivates this method. There is a universal challenge at the heart of chemistry and biochemistry, which is due to an unbridged gap between chemical insight and quantum mechanics. This challenge is best explained by a representative example (see below) but it essentially states that *we must be able to detect, by computation, which fragment (atoms, functional groups) of a given molecular assembly governs the energetic behaviour of this whole assembly*. If solved, this quantum-based insight will rigorously guide predictions on the relative stability of molecular assemblies. REG, which will be explained at the end of this section, is a promising attempt to tackle this challenge. So far all REG case studies have been carried out in conjunction with IQA but REG could in principle operate with non-IQA atomic energies or even other atomic properties.

As announced, an example will sharpen the nature of the challenge described above. Textbooks state that the guanine…cytosine complex is more stable than the adenine…thymine one because the former is held together by three hydrogen bonds while the latter by only two. In this case, the atoms in the hydrogen bonds constitute the fragment that is supposed to govern the energetic stability of the complexes. Yet, the uracil…2,6-diaminopyridine complex, which also has three hydrogen bonds, is two orders of magnitude less stable than guanine…cytosine. Clearly, counting hydrogen bonds does not explain stability. A (bio)chemist wants to understand the behaviour of a system by the presence or action of relevant atoms in the system, i.e. a “localised explanation”. However, this undertaking can derail, as shown in this well-known example, and thereby undermines confidence in standard chemical intuition.

However, in order to remedy the inadequacy of focusing on hydrogen bonds only, Jorgensen and Pranata proposed [145] their secondary interaction hypothesis. This hypothesis is actually a simple rule, based on more distant atomic interactions than those involved in the hydrogen bonds only. This rule claims to make reliable predictions of stability, and again on the “back-of-an-envelope”, like hydrogen bonds claim to do. However, this proposal did unfortunately still not solve the main problem of how and why a molecular assembly is held together, in terms of key atoms. Sure, the secondary interaction hypothesis was seized by supramolecular chemists (e.g. Gellman, Zimmerman, or Rebek) as the next best concept beyond hydrogen bonding, to explain and predict relative stabilities of various complexes. However, the hypothesis fails [146–150] regularly. Furthermore, it was shown that the secondary interaction hypothesis cannot be linked to the underlying quantum reality offered by modern wavefunctions. In other words, if this rule works on the right occasions, it does not do so for the right reasons.

This unsatisfactory state-of-affairs prompts one to look harder to find that much needed, reliable bridge between modern wavefunctions and back-of-an-envelope explanations. This bridge is not just essential for the pivotal example given above but also for a wide variety of recurring questions such as:

- what is the origin of this torsional rotation barrier? [151]
- what holds heterocyclic aromatics (including DNA base pairs) together? [152]
- what is the degree of covalency [153] of this halogen bond?
- which atoms are actually driving this reaction [154] and by which energy type?
- what is the character of this mysterious through-space interaction [155] in this enzyme?
- how is this molecular crystal held together in the curious absence [156] of hydrogen bonds?
- why is the distance between these two atoms suspiciously short, again and again? Is there perhaps a new type of non-covalent interaction [157] ?
- if the fluorine gauche-effect is actually electrostatic in nature [158] (unlike what Wikipedia claims) then how does this knowledge influence the future design of compounds in terms of highly desirable conformational control?

**Fig. 7:**
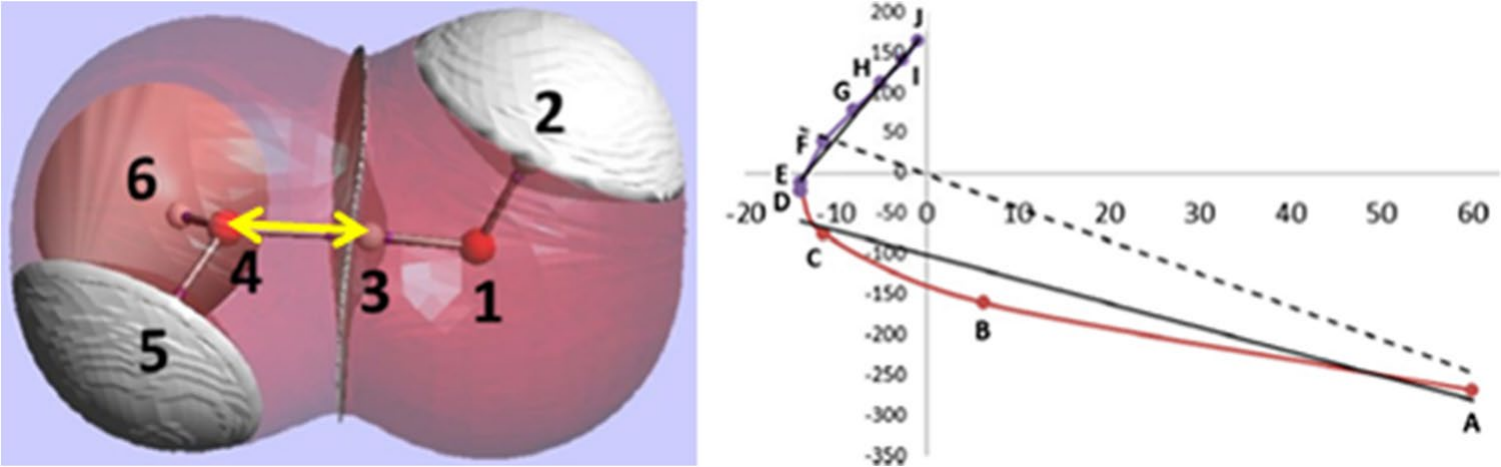
(Left) The arrow represents the control coordinate governing the REG for the water dimer; (right) the correlation between the total energy of the dimer (*y*-axis) and the electrostatic interaction energy V_elec_(H_3_,O_4_) (*x*-axis). The dashed line shows the poor linear fit if the whole curve is considered while much better correlations are obtained after segmentation into a purple and a red curve*.* Note that all energies were “translated”; i.e., their respective mean energies, averaged over the various lettered geometries, were subtracted

So how does REG work [134] ? The method is dynamic: its control coordinate *s* (yellow hydrogen bond length in Fig. 7) induces a geometry change in a molecular system (e.g. motion along a reaction coordinate, torsional rotation. In this case, this change is a compression of water monomers in a water dimer).

The total energy of the molecular assembly (*n* atoms) is made up as follows,

11 $${E}_{tot}\left(s\right)=\sum_{i=1}^{N}{E}_{i}(s)$$

from *N* atomic energy contributions E_i_, and $\mathrm{N}=2\left[\frac{\mathrm{n}\left(\mathrm{n}-1\right)}{2}\right]+\mathrm{n}={\mathrm{n}}^{2}$. An example of E_i_ is V_elec_(H_3_,O_4_) of Fig. 7. The value N is calculated like this because there are $\frac{\mathrm{n}\left(\mathrm{n}-1\right)}{2}$ unique pairwise interaction energies of the type V_X_(A,B) and again $\frac{\mathrm{n}\left(\mathrm{n}-1\right)}{2}$ of the type V_elec_(A,B), while there are n intra-atomic energy contributions E_intra_(A). So, for the small system that is water dimer, the number of energy contributions is already 36. The right panel of Fig. 7 shows that the correlation between *E*_*tot*_ and an *E*_*i*_ value is best fitted by least-squares,

12 $${E}_{i}\left(s\right)={m}_{REG,i}{E}_{tot}\left(s\right)+{c}_{i}$$

and in two energy segments: (i) in the dimer formation the slope m_REG_ is highest of all E_i_, i.e. for the electrostatic energy between H_3_ and O_4_ denoted V_elec_(H_3_,O_4_), and (ii) in the dimer compression the internal steric energy O_4_ has the highest slope. The Pearson correlation coefficient must obey | R_i_ |> 0.95 in order to be meaningful. The dashed line in Fig. 7 is thus not meaningful and the dimer should be analysed in two energy segments rather than in one. In a proof-of-concept case study [155] on a protease enzyme, REG managed to isolate, from a ~ 130 atoms active site with ~ 17,000 energy terms, the concerted bond breaking/making mechanism of the substrate’s hydrolysis. REG also identified and quantified O…O through-space interactions with some covalency.

We close this section with a colourful metaphor that allows one to grasp the essence of what REG strives to achieve. Let us make a comparison with analysing a football match. An atomic energy equates to a player and the total system’s energy equates both teams. The actions of all players are “added up” to yield an overall football game, which is similar to the overall energy profile. Now, the scoring of a goal is a particular phenomenon occurring in the game (i.e. the molecular system changing dynamically). This goal corresponds to a chemical phenomenon, for example, two molecules forming a stable complex. The question is now how one can *explain* this goal. Does one look at each player’s contribution, no matter how small, and distribute the “explanation” over all their actions? Or does one give up on finding the explanation altogether and simply say that the goal emerged somehow from both teams interacting? Or does one focus on the last action of one or two players only, just before the goal, fast and dramatic as it was? The answer is typically “yes” to the last option. Equally, *REG reveals the “player” who made that goal*, where the fast, last-second action corresponds to the highest-ranking REG value. The REG method can reveal the player even if other players tried to prevent the goal. More formally, REG isolates the atomic energy that has that highest ratio between its own dynamic slope and that of the total system. In other words, REG finds out which atoms drive the change in the total system the hardest.

### 2.13 Electron correlation energy

The topological energy in Eq. (5) that is the most difficult and time-consuming to compute is ${V}_{\mathrm{ee},\mathrm{c}}^{AB}$ or simply ${V}_{\mathrm{c}}^{AB}$. Although values were already calculated at full CI level for H_2_ and He_2_ in the original IQA paper [44] of 2005, MP2 (as well as MP3 and MP4SDQ) energies only became available [80] in 2016 for the first time. Even more recently, others have proposed [159] an efficient implementation of the MP2 energy, with a nearly linear scaling with respect to the number of basis functions. This work already enabled modest computing times to obtain ${V}_{\mathrm{c}}^{AB}$ values for various classical configurations of ethane dimers, and opens an avenue towards more elaborate studies of non-covalent interactions. Our own work [160] led to a dramatically faster and more accurate calculation of ${V}_{\mathrm{c}}^{{AA}^{^{\prime}}}=\sum_{B}{V}_{c}^{AB}$(where index *B* is allowed to be equal to *A*, and *A*′ stands for the environment of *A* and *A* itself) for MPn (n = 2,3,4) wavefunctions by introducing analytical integrals reminiscent of the electrostatic potential. Having only ${V}_{\mathrm{c}}^{{AA}^{^{\prime}}}$ values means that pairwise interactions are not known. However, this shortcoming does not harm the development of FFLUX, which only uses ${V}_{\mathrm{c}}^{{AA}^{^{\prime}}}$ anyway. In 2015, IQA became linked [161] with coupled cluster theory, and in 2020 our own CCSD-IQA version [162] made it possible to compare its ${V}_{\mathrm{c}}^{AB}$ values with MP2-based ones.

We noticed a rule when studying [163] water clusters (up to pentamer) with IQA at MP2/6-31G** level. An oxygen that does not accept a hydrogen has an intra-atomic correlation energy of − 487 kJ mol^−1^ with a standard deviation of only about 1 kJ mol^−1^. When this oxygen accepts one hydrogen (via a hydrogen bond) then its energy decreases by 8 kJ mol^−1^ (to a more stable value of -495 kJ mol^−1^). When this oxygen accepts a second hydrogen, then its energy decreases again, by another 8 kJ mol^−1^. This tight clustering and additivity is a sign of high transferability, a general hallmark of QCT.

**Fig. 8:**
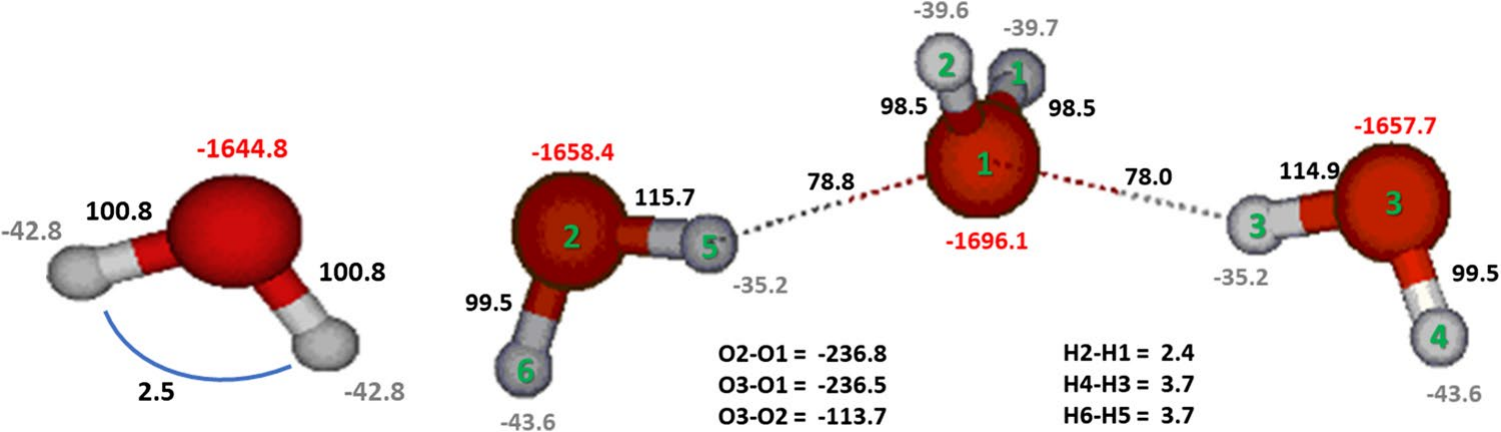
A variety of inter-atomic CCSD-HF correlation energies (kJ mol^−^.^1^) in a linear configuration of the water trimer (H_2_O)_3_, including both intramolecular and intermolecular (values in black for both), as well as intra-atomic correlation energies (values in red for oxygen and grey for hydrogen). Nuclei are labelled in green. For comparison, a single free water is represented on the left

There exists a remarkable degree of transferability of CCSD-HF correlation energies in water clusters. Compared to Møller-Plesset, CCSD values show a higher sensitivity and are now also able to pick up if an oxygen donates a hydrogen, from this oxygen’s intra-atomic energy. Indeed, its energy drops by ~ 15 kJ mol^−1^ while donating a hydrogen and by ~ 25 kJ mol^−1^ while accepting a hydrogen. Moreover, these two decrements are additive, and explain the changes of the intra-atomic correlation energy of all oxygens in Fig. 8 (and others not shown), compared to that in a single water. Figure 8 shows inter-atomic correlation energies, both intra-molecular and intermolecular, as well as intra-atomic ones, all computed with the uncontracted 6–31 +  + G(2d,2p) basis set.

Indeed, the central oxygen in the linear trimer accepts two hydrogens and should therefore have an energy of − 1645 + 2x(− 25) =  − 1695 kJ mol^−1^. The two oxygens in the dimer (not shown) are also nicely predicted. The intra-atomic energies of the hydrogens also make sense: when “free-standing” (i.e. single water or a water dangling of a complex) the value is − 42 ± 1 kJ mol^−1^ while, when in a hydrogen bond, it is − 33 ± 2 kJ mol^−1^. Equally convincing tight clusters are found in the interatomic correlation energies (both intra-molecular and intermolecular).

An analysis of a deprotonated glycine…water complex calculated at uncontracted MP2/6-31G** level showed [81] that the nonbonded interactions have abundantly negative ${V}_{\mathrm{c}}^{AB}$ values. The most negative of these nonbonded interactions are the intramolecular H…H interactions, generally around − 5.0 kJ mol^−1^. However, notable exceptions are interactions between non-hydrogen atoms, which can be surprisingly large, such as the O…O correlation (11.6 kJ mol^−1^), which is about the same value as that of the C = O bond (10.1 kJ mol^−1^). The hydrogen bonded O…H correlation value is − 3.5 kJ mol^−1^. A collection of hydrogen-bond results [164] shows that, at MP2 level, there is a remarkable near-cancellation in electron correlation energies between the negative H…X value and the positive X′…X values in [X′-H…X] systems spanning NH_3_…H_2_O, HF…H_2_O, Gly…H_2_O and (H_2_O)_n_ (n = 2 to 5),

Calculations on the D_2d_ configuration of the ethene dimer give a glimpse of π…π interactions, where no intermolecular interactions were larger than 1.0 kJ mol^−1^. The net value of the intermolecular values is only − 2.8 kJ mol^−1^, which is small but roughly of the same magnitude as the total stabilisation energy of the ethene complex, − 6.5 kJ mol^−1^, calculated at the MP2/CBS level of theory.

### 2.14 A final note on FFLUX

Many of this article’s ideas, provocative as they may be, actually follow from the informed choice of using quantum topological atoms. If they are at the heart of how to look at atomic behaviour and interactions then they come with consequences. Although this is an article on non-covalent interactions and not on force field design, FFLUX permeates much of the article’s core text and the external discussion following it. Thus, it is deemed useful to clarify three more points.

First, QCT is not a method for solving the Schrödinger equation but a theory (and methodology) that enables the (chemical) interpretation of modern wavefunctions. QCT thus acts a robust bridge between quantum mechanics and chemical concepts. Like conventional EDA methods, QCT cannot predict intermolecular interactions in the way that SAPT can. SAPT is an accurate method of computing energies of non-covalent interactions while QCT is not. Moreover, SAPT offers an interpretation, be it more convoluted than that of QCT. Yet, FFLUX has predictive power, by using its machine-learnt atoms to predict the structure and dynamics of single molecules or assemblies thereof. These atoms have captured the knowledge (of energy and multipole moments) from QCT data, and make it available, essentially through high-dimensional interpolation.

Second, one may have the impression that FFLUX is predominately on the drawing board. Indeed, this article has not systematically reported on all its case studies because this is not the scope of the article. It is true though that FFLUX is not yet routinely used and its applications are still in the realm of development. All work has focused on reaching proofs-of-concept and on software implementation, given that FFLUX was created from scratch (or ab ovo if one wishes).

Third, the one remaining proof-of-concept to be reached is that of dressing up a large system with machine learning models prepared for fragments of that system. This step necessitates invoking transferability. Fortunately, quantum topological atoms score highly on this front and thus the hypothesis will very likely be successful. This route will enable FFLUX to make predictions for peptides and even proteins in aqueous solution, a long envisaged goal. The training procedure of FFLUX can already handle systems of ~ 70 degrees of freedom thanks to the paucity of data points needed. This is because we use adaptive sampling (also known by the fancier name of active learning), and also because Gaussian process regression needs fewer data points than neural networks do. This is how FFLUX is expected to cope with the dimensionality curse. Of course, degrees of freedom can be frozen in a controlled manner, in order to deploy them where they are needed. For example, the ψ and φ dihedral angles are much more important in covering secondary structure than a C_α_-H bond length, for example.

## 3 Conclusions

This perspective proposes a view on non-covalent interactions that is inspired by the (quantum) topological approach and its consequences. The 14 subsections can be summarised as follows, a numbered statement for each:

1. Five arguments aim to justify the topological partitioning as a way to define an atom inside a system in spite of its high computational cost relative to non-topological methods.
2. All atomic properties come from a single, universal volume integration. The concomitant energy terms (IQA) are mathematically defined and interpreted in terms of their chemical meaning.
3. Covalency is a sliding scale rather than a binary assignment of covalent versus non-covalent, and “through-space” interactions can be properly characterised. Hydrogen bonding is seen as a three-atom phenomenon (donor-hydrogen…acceptor).
4. The full consequence of the non-overlapping nature of topological atoms is deliberated, empty space and associated energy leakage correctly lacking. An example shows how two molecules forming a van der Waals complex do not overlap but instead indent or distort each other.
5. How the dichotomy between bonded and non-bonded interactions affects the energy architecture of classical force fields is explained. The absence of this dichotomy in the topological energy partitioning opens an avenue for the design of a novel type of force field called FFLUX.
6. Reasons are given why FFLUX sidesteps perturbation theory in the context of the latter’s more general shortcomings, and how its treatment of polarisation does not enforce the rigid body constraint.
7. How the space-filling nature (no overlap, no gaps) of topological atoms avoids penetration effects and damping functions is rationalised.
8. The role of atomic monopole moments is discussed. It is argued that atomic multipole moments are the most promising way forward to improve the current status quo of inherently limiting point-charge electrostatics. Many systems are made up of polar molecules, which thus need accurate electrostatics to model their non-covalent interactions. Topological monopole moments should not be seen as another way of obtaining point-charges but only as a measure of charge transfer. The latter is only one of two types of contributions to the molecular dipole moment, and can thus not be used by itself to assess how topological monopole moments reproduce molecular dipole moments.
9. Details are given on the convergence behaviour of multipolar electrostatics formulated in the spherical tensor formalism. Exact formal convergence is possible (and does occur) but often a plateau enables practical convergence. Which multipole rank is necessary to reach a given energy accuracy for two given elements at a certain distance is well understood.
10. A way of handling polarisation is explained, which uses machine learning to focus on the end point of the polarisation process rather than the process itself. Essentially, charge transfer is the effect of “monopolar polarisation”. Thus, charge transfer is not in need of special handling but is just another special case of multipolar polarisation.
11. Bond critical points are discussed, with a brief characterisation and history, culminating in serious concerns about their ultimate meaning. This in spite of an interesting link between their appearance and an observed balance of interatomic exchange energies, and in spite of the likely settling of an old controversy on their meaning in biphenyl (H_ortho_…H_ortho_).
12. A novel method called Relative Energy Gradient (REG) is proposed, which is able, by computation, to detect which fragments of a given molecular assembly govern the energetic behaviour of this whole assembly. Applications in the area of non-covalent interactions are given (e.g. halogen bonding, hydrogen bonding, DNA base pairs, torsional rotation barrier, and the fluorine gauche-effect and (enzymatic) reaction).
13. An analysis of electron correlation energies shows remarkably tight clusters of intra- and intermolecular energies supplemented with additivity. These are both hallmarks of high transferability, found both in CCSD and MPn (n = 2,3,4) wavefunctions of water clusters. A few simple rules enable predictions on the back of an envelope.
14. FFLUX has predictive power, unlike IQA or QCT. This force field has been designed from scratch, which explains why it is mainly in the stage of having reached proofs-of-concept. With all strands coming together, it is well poised to exploit IQA’s high transferability to finally predict the structure and dynamics of peptides in aqueous solution.

## External discussion

### Abstract

#### Mo, Danovich, and Shaik ask:

The author outlines the architecture of a force field called FFLUX, which is based on these ideas. Furthermore, a new method, so-called Relative Energy Gradient (REG), is introduced. REG uses computation, to detect which fragments of a given molecular assembly govern the energetic behaviour of the whole assembly. According to the author, this method can offer insight in the typical balance of competing atomic energies both in covalent and non-covalent case studies. Can the author explain this statement clearly to the reader who is not conversant in this theory?

## Reply:

The quoted sentence appears in the Abstract. While it may be somewhat cryptic there, the novice reader best starts with the football metaphor explained in the last paragraph of Section 2.12. This metaphor sets the scene for thinking about what happens a lot in chemistry: a near cancellation of relatively large and opposing energy variations. From the net energy change then emerges a chemical effect characterising the whole system. An instructive example is the case study of the rotation barrier in biphenyl, [133] which is discussed in Section 2.11. At the level of total atomic energies, REG identifies the energy behaviour of the C_ortho_ atoms as the cause of the planar barrier. These atoms are actually the “third dog that carries away the bone” to use another metaphor. The two dogs that are fighting are: (i) the (two) *ortho*-hydrogens (which are destabilised, equivalent to the textbook steric clash) and (ii) the formation of a weak covalent bond between the *ortho*-hydrogens.

## Introduction

### Szalewicz comments:

I would disagree with the statement: “it will probably take a long time before scientists meet the challenge of both quantitatively predicting and qualitatively understanding non-covalent interactions”. Of course, computational predictions will always be restricted by the sizes of systems, however, such predictions are possible for quite large systems. For example, in a recent paper from our group [165] we present benchmark calculations at the complete basis set limits for dimers containing hundreds of atoms. These results compare very well with results extracted from experiments. Another example are our group first-principles predictions of properties of water, [97] from water dimer spectra to properties of liquid water. All work from our group that uses symmetry-adapted perturbation theory (SAPT) automatically provides qualitative understanding of intermolecular interactions, as detailed in the paper which is a part of this Conversation.

### Reply:

My statement refers to the totality of the literature, not an individual pockets of excellence, which I will be the first to appreciate and respect. Sure, SAPT has made future-proof contributions to be celebrated but its impact in force field design and application, for example, is far from complete. Force fields still use predominately unpolarised point-charges alongside Lennard–Jones potentials with which they study how proteins interact with water, systems much too large to study directly with SAPT. Having looked at various research fields focusing on non-covalent interactions, I find that they do not link up well, if it all. For example, the highly accurate ab initio work of Sherrill on benzene dimer configurations (the typical study on parallel, slipped parallel and stacked geometries) does not merge with the work of Hunter who explains the stability of assemblies of aromatic rings differently, coming from a tradition of experimental supramolecular chemistry. Equally, the advancing experimental field of molecular balances aims at explaining the nature of the forces holding molecular fragments together but currently lacks a convincing and indeed necessary theoretical/computational backup at atomistic level. Would exquisitely accurate work on noble gas dimers or very small van der Waals complexes (e.g. Meath, Aziz, Hutson) help here? Going beyond this example (rooted in physical organic chemistry) to mesoscopic modelling, the popular coarse-grain force field MARTINI made design decisions that are hard to justify in the light of interpretative quantum chemistry. The list goes on if one includes the ubiquitous and implicit (rather than explicit) solvation model COSMO-RS or the protein modelling package Rosetta, which contains fudge factors and a tenuous link to SAPT, for example. In summary, I believe that we do not yet have a consistent, accurate and fully linked up, predictive framework to correctly describe non-covalent interactions in sizeable condensed matter systems, never mind that we can explain hydrophobicity with confidence, to name another important topic.

## Section 2.1

### Szalewicz asks:

Is it indeed true that “many will agree that non-covalent interactions are typically described at atomistic level”? Quantum mechanics is a holistic theory. Schrödinger’s equation for a molecule does not contain only atomic terms, it has interatomic ones as well. SAPT has to use molecules as unperturbed systems, it cannot start from a set of atoms. One can use methods such as QCT to partition interaction energies into atomic contributions, but this can be only done after some holistic QM calculations are performed (QCT alone cannot predict any molecular properties).

### Reply:

Quantum mechanics is holistic in the sense of quantum entanglement but the usual Alice and Bob signalling between two galaxies is less relevant to quantum chemistry. The principle of locality in chemistry means that a perturbation on one side of a microscopic piece of matter does not cause changes a macroscopic distance away. My statement was meant to reflect on the resolution with which one attempts to explain non-covalent interactions. One can think of at least four levels of resolution starting with the coarsest: (i) molecules, (ii) functional groups (or unnamed molecular fragments), (iii) atoms and (iv) subatomic features (e.g. lone pairs). The atom may play a central role (because this is where Chemistry starts, as it were) but it can lead to too fine a resolution. In its original form, SAPT cannot offer submolecular explanations although Sherrill and coworkers recently proposed A-SAPT. Due to serious shortcomings they quickly replaced by A-SAPT by F-SAPT.

### Misquitta asks:

Surely we expect AIM charges to vary in some systematic way with changes to chemical environment, and also with conformation. Are you saying that they should not, or that they should vary in *some* way, but perhaps not by much? If so, how would we judge which charge model is better? And hence how would we know how much change is just right?

### Reply:

In the main text I did not have space to discuss the two different matters behind the statement that “QTAIM is … not too sensitive to details in the electronic structure calculations from which they are derived (basis set, conformational changes, chemical changes in the environment)”. One matter is basis set dependence. Ideally, a charge is not very dependent on the nature of the basis set but Mulliken charges have been known for decades to be highly unstable. I am referring to very large changes, and even sign flips. The presence of diffuse functions causes wild fluctuations also in the charges according to Distributed Multipole Analysis (DMA), [17] at least in its original formulation. Based on their extensive study [166] of several atomic charges by several criteria Wiberg and Rablen concluded that they prefer (QT)AIM charges over any other.

In terms of the second matter, on conformational change and chemical change in environment, the latter change has also been looked at by Wiberg and Rablen. These authors studied a few dozen small organic molecules with the following methods: Mulliken, NPA, Hirshfeld, AIM, GAPT and CHELPG. They state that “the CHELPG charges at first appeared attractive in that they reproduce the molecular dipole moments and electrostatic potentials. However, in view of the lack of response to changes in electronegativity, and the problems noted with the hydrocarbons, they cannot be recommended for studies of substituent effects or other intramolecular interactions.” This is an example of a charge that cannot be rolled out in the context of transferability, which is the 0^th^ cornerstone of force fields for macromolecules. Secondly, the reason why charges that are least-squares fitted to molecular electrostatic potentials (such as CHELPG) are unstable with respect conformational changes, is well explained by Francl et al. [167] (in terms of matrix rank deficiency) and further discussed [70] in the framework of drug design.

### Misquitta asks:

The ISA is also a ‘minimal’ approach in a well-defined sense. No references are needed, and atoms are made as spherical as possible in a well-defined mathematical sense. Would you therefore include the ISA as satisfying Occam’s razor?

### Reply:

It is true that the lack of a reference makes ISA minimal but introducing (imposing?) spheres does not. In my opinion, the latter is a hallmark of simplicity, not minimality. The manuscript hints at my distinction between minimal and simple as discussed in the preface of ref. [27]. It is useful to repeat this clear example explaining the difference. The ancient Greeks thought of the heavens as perfect: planets moved in circles, which are objects of high symmetry and thus beauty and perfection. But then, deviations of this circular motion were observed. Holding on to the idea of a circle, the Ptolemaic system (amongst others) proposed epicycles to explain this observation. This strategy is *simple*: it imposes the wrong idea onto a natural phenomenon, guided perhaps by a misplaced sense of beauty. On the other hand, a *minimal* explanation is the ellipse. One needs only one ellipse to describe the planetary orbit rather than many circles (epicycles) and thus one complies with Occam’s razor. The ellipse is “uglier” than the circle, and indeed more general (having two radii), but in one fell swoop it captures the essence of the natural phenomenon (i.e. the orbit). Its minimality is expressed in Kepler’s laws, which were later shown to be compatible with Newtonian mechanics. I do not think that one should insist on making atoms spherical if they sit inside a system. Hence, ISA does not satisfy Occam’s Razor in full.

### Misquitta asks:

Please explain what you mean by "The smallest deformations are found in space-filling decompositions, which generate a less distorted image of chemical phenomena leading to smaller deformation and interaction energies. This is an important conclusion because it is at the heart of chemistry…”.

### Reply:

Some numerical details behind this statement can be found in ref [30]. but even more so in a reference, [168] which was not mentioned in the main text. Before answering the question some background is necessary. Real-space (i.e. non-Hilbert-space) atomic partitionings, such as the quantum topological one or the Hirshfeld one, can be unified under one simple scheme. The system’s electron density ρ(**r**) can be written as the sum of atoms-in-the-system density ρ_A_(**r**) as follows:

$$\rho \left({\varvec{r}}\right)=\sum_{A}{\rho }_{A}\left(\mathbf{r}\right)=\sum_{A}{w}_{A}(\mathbf{r})\rho \left(\mathbf{r}\right)$$

where w_A_(**r**) are atomic weight functions that satisfy $\sum_{A}{w}_{A}\left(\mathbf{r}\right)=1 \ \forall \mathbf{r}$.

There are several possible partitionings of unity (i.e. summing up to 1), and the one corresponding to quantum topology is defined as

$$w_A\left(\mathbf r\right)=\left\{\begin{array}{l}1\;\;\;if\;\mathbf r\;\in\Omega_A\\0\;\;\;\mathrm{elsewhere}\end{array}\right.$$

while definitions of others can be found in the reference above. The deformation energy E_def_ for an atom *A* is defined as ${E}_{def}^{A}={E}_{\mathrm{intra}}^{A}-{E}_{0}^{A}$ where ${E}_{0}^{A}$ is the energy of the atom *in vacuo*.

Now, to answer the question about what “smallest deformation” means, it was established that QTAIM atoms tend to give the smallest overall deformation energies, followed by the less diffuse Becke(T) atoms, while the diffuse atoms of Hirshfeld and MinDef (“Minimal deformation criterion” [169]) usually give quite large deformation energies.

### Misquitta asks:

I understand this philosophical stance of the final paragraph of Section 2.1 but surely there are just as many examples from Life of systems which do inter-penetrate: ecosystems have no hard boundaries, nation states like to impose hard boundaries but these are almost never respected by the cultural groups that more often than not freely interpenetrate.

### Reply:

Sure, this is true. However, my point was never that all of Life is sharply bounded or “non-overlapping”. I just brought up a number examples in the natural (and human) world where sharp boundaries are well recognised. Indeed, people can move in and out of nation states, in the same way electrons can move in and out of topological atoms. Yet the nation states themselves (like topological atoms) do not overlap. In fact, in an earlier publication (Fig. 4 in ref [72]). I have compared a topological atom with a stationary water pattern in a flowing river.

## Section 2.2

### Szalewicz comments:

The two-body spin-integrated density ρ_2_(**r**_**1**_, **r**_**2**_), called in the paper “diagonal second order reduced density matrix” (perhaps it should be defined to avoid confusion), is partitioned in Eq. (5) into three parts: product of one-body densities, product of one-body density matrices, and the remainder called the correlation part. These parts upon integration with 1/r_12_ lead to components called Coulomb, exchange, and correlation, respectively, related to various intermolecular interaction components in Fig. 2. I would like to point out that, except for the Coulomb term, this terminology does not agree with that used in SAPT. The SAPT exchange energies can be formulated in terms of density matrices, see ref., [170] but of different types. Similarly, in SAPT terminology a part of electron correlation is included already in the one-body densities and density matrices. Thus, EDA based on Eq. (5) has to disagree with SAPT and most other EDAs.

### Reply:

This comment does not pose a question other than defining the diagonal second order reduced density matrix:

$${\rho }_{2}\left({\mathbf{r}}_{1},{\mathbf{r}}_{2}\right)=N\left(N-1\right)\int {ds}_{1}{ds}_{2}{d\mathbf{x}}_{3}\dots {d\mathbf{x}}_{N}{\Psi }^{*}({\mathbf{x}}_{1},{\mathbf{x}}_{2},..,{\mathbf{x}}_{N})\Psi ({\mathbf{x}}_{1},{\mathbf{x}}_{2},..,{\mathbf{x}}_{N})$$

where *N* is the number of electrons, **x**_i_ the spatial and spin coordinates of the i-th electron, and **r**_1_ and **r**_2_ span a 6D space. The integration over spin variables s_1_ and s_2_ is actually a summation. Note that the prefactor N(N − 1) follows the convention of McWeeny, which is different to that of Löwdin and Bader who both divide this factor by 2. We define it here rather than in the main text in order not the break its flow. Furthermore, extensive comparisons and commentaries on SAPT, and energy partitioning methods can be found in a fairly recent chapter [171].

### Mo, Danovich, and Shaik ask:

Recently, a few approaches like IQA have been developed within the framework of QTAIM. QTAIM and its descendants (generally called, the Quantum Chemical Topology (QCT) approach as suggested by the author who is using QCT here for the discussion of non-covalent interactions. In this highly intense perspective where readers are assumed to know QCT in details (do they really?), the author advocates the concept of space-filling atoms which is different from the conventional ball-and-stick picture of molecules. This is an interesting concept. Nevertheless, this and the subsequent energy partitioning are purely hypothetical. What are the advantages of these definitions? Are they universally effective? Can the approach provide insights into the nature of intra- or inter-molecular interactions? E.g., there are controversies over the nature of ethane rotation barrier with two main explanations concerning either hyperconjugative attraction or steric repulsion. Can the QCT analyses offer opinions on this kind of controversies?

### Reply:

I am sorry if not enough details were given in this article, which was already longer than the guidelines recommended. However, didactic accounts can be found in the 4 chapters I wrote (refs [3–6]).

Any energy partitioning is hypothetical, which is maybe why there are so many of them and why researchers cannot agree on which one to prefer. This situation caused Section 2.1 to go to great lengths in listing the (at least) 5 advantages of the topological partitioning. They are quickly summarised as: (i) the atom can be obtained, through the electron density, from SCF-LCAO-MO, X-ray diffraction or grid-based schemes, and atomic charges are stable with respect to basis sets; (ii) minimality: no parameters needed, just the gradient path; (iii) space-filling decomposition better preserves atomic identity in terms of energy; (iv) well-defined kinetic energy; and (v) a deep connection with quantum mechanics. These QCT advantages also benefit IQA and thereby answer one of the questions asked above.

Yes, the advantages are universally effective in that IQA can be used in any system: organic, inorganic, biochemical, materials, clusters, liquids, solids, etc. Indeed, IQA offers insight as demonstrated in the 2020 Review given in ref. 45. Ethane has been investigated by IQA more than a decade ago [62] where the hyperconjugative interpretation was supported. When combined with the Relative Energy Gradient (REG) method, IQA can indeed give a clear opinion on controversies, such as the one on the rotation barrier in biphenyl [133]. However, A REG-IQA analysis has not yet been carried out on the rotation barrier in ethane.

### Mo, Danovich, and Shaik ask:

In Section 2.2, the author cited ref. 58 by stating “the overall intra-atomic energy has been fitted successfully to the repulsive part of the Buckingham potential for van der Waals complexes”. We assume that readers may wonder how intra-atomic energy can be related to the inter-atomic energy. Figure 2 shows the “conceptual” correlation of IQA energy terms with specific chemical insights, which, unlike other energy decomposition schemes such as the mentioned Kitaura-Morokuma analysis, cannot be quantified but loosely related to their objectives. Unless theoretically proved, such kind of correlations cannot be over-interpreted. Can the author comment on this issue?

### Reply:

I think that there are two quite different parts to this question: (i) the surprising intra-atomic nature of repulsion, and (ii) the link between IQA energies and chemical concepts.

Firstly, the initially unexpected intra-atomic nature of repulsion can be justified in some way. It is true that it is usually described as an interatomic interaction. Indeed, the ubiquitous Lennard–Jones potential, for example, portrays the repulsion between two atoms as having a r_AB_^−12^ dependence where the internuclear distance r_AB_ emphasises the interatomic nature of the interaction. There is, however, no theoretical proof of this law. In fact, ref [58] confirms that, by using IQA energies, the exponential law of the Buckingham potential fits better than r_AB_^−12^_._ Now, a pivotal component of the repulsion energy “between” two atoms is their respective kinetic energies. Upon compression, the kinetic energies rise dramatically and the two potential energies, ${V}_{en}^{AB}$ and ${V}_{nn}^{AB}$ (which also contribute to the overall intra-atomic energy) largely cancel. Kinetic energy is manifestly associated with a one-electron operator and can therefore not be expressed as an inter-atomic quantity. Thus, the repulsion energy is mono-atomic or intra-atomic energy is nature. Note that our work in ref [80] numerically showed that, in He_2_ for example, electron correlation is much larger within the heliums (or helia) than between the heliums. This observation suggests that for van der Waals dispersion, correlation energies represent an atomic stabilisation, by proximity to other atoms, as opposed to direct interactions with other nearby atoms. This situation is thus similar to that of repulsion. However, in the case of electron correlation the conceptual image we arrived at is similar to that of Feynman proposed in 1939.

Secondly, Fig. 2 is backed up by many IQA-based papers, and the mapping between the 3 terms and chemical concepts is well established from a practical and observational point of view. Hence I do not believe that this connection is loose; instead, it is highly quantitative and may even help sharpening the definition of a chemical concept.

### Mo, Danovich, and Shaik ask:

Altogether, this Perspective is a condensation of many works and sometimes it is quite difficult to understand or follow. For example, in the last paragraph of Section 2.2, the author wrote: “it is possible to lump the intra-group (deformation) energy into the inter-group energy. There is no unique way of doing this but a popular choice is using the ratio of an inter-group energy to the sum of inter-group energies as a weight that contributes to the intra-group energy”. But from the second sentence, it seems to “lump the inter-group energy into intra-group energy”. In Secion 2.3, we think that the N O interaction in the O = C-N group is not “through space” but “through bond” due to the π conjugation. Can the author respond to these concerns?

### Reply:

I tried to be as clear as possible but yes, much work is coming together here and the article is already long. I believe that the text in Section 2.2 is correct. There is no contradiction when inspecting Eq. (9) of ref [49] that I was referring too. However, note that there are two errors in this equation: the superscript in the middle term E_def_ should be “G” rather than “I”, and the prime in the second line should be near “E” rather than “H”.

I did not consider the πconjugation, which is a good idea. In that case, one can indeed speak of “through bond”. However, the more general point of this section does refer to “through space”, in the absence of conjugation.

### Misquitta asks:

In Section 2.2 there is nothing in the IQA partitioning that is reliant on the hard boundaries of the QTAIM approach. Surely all the integrals presented here could just as well have been expressed via the smooth, interpenetrating atoms of the H-I, ISA, MBIS approaches. Could you comment on this possibility in the paper as this seems like an important point?

### Reply:

This is correct: my answer to another of your questions briefly reviewed the unified “partitioning of unity” scheme. IQA is based on McWeeny’s theory of electronic separability, and its use of density matrices can be combined with the “unity scheme” such that all approaches mentioned above can tap into equations such as Eqs. (2), (3) or (5), for example. There is one exception, in connection with Eq. (4), which involves the (atomic) kinetic energy. Within QCT (or QTAIM or IQA) the atomic kinetic energy is well defined (see ref [32]. and refs. therein) (at least within the Laplacian family of local kinetic energies) thanks to the nature of the zero-flux surfaces bounding the topological atoms (using Gauss’s divergence theorem). This advantage does not apply to the non-QCT methods mentioned above.

## Section 2.3

### Brinck and Borrfors ask:

The concept of a sliding covalent scale on page 8 is very interesting. However, we would have liked to see some concrete examples of the scaling for intermolecular interactions where the covalent contribution has been debated. For example, could you provide some comparisons between hydrogen bonded and halogen bonded complexes where the acceptor molecule is the same? How does the covalent index of a strong donor acceptor system, e.g. BF_3_ •••NH_3_ compare to a traditional covalent single bond, e.g. B-F or N–H, in the same system? It would also be interesting to see a distance dependent analysis of the covalent indicator for some systems.

### Reply:

In ref [67] there are many more systems, other than the water dimer, where the “covalent contribution has been debated” but not discussed in Section 2.3. All energies to be quoted have been calculated at HF/6-311G(d,p) level.

As a first example, the C_ipso_-C_ortho_ interaction amounts to 1097 kJ/mol in terms of covalent strength (|V_X_(A,B)|), while the C_ipso_-C_meta_ through-space interaction amounts to only 26 kJ/mol, which is ~ 40 times weaker. Naively one would expect the C_ipso_-C_para_ through-space interaction to be even feebler given the longer internuclear distance. However, this interaction amounts to 24 kJ/mol, which is about the same strength as for C_ipso_-C_meta_. This can be explained by the conjugative stabilisation (i.e. delocalisation) over the whole benzene ring. This is why C_ipso_-C_meta_ is perhaps best called a through-bond interaction. In any event and in summary, there is extra stabilisation occurring between pairs of carbon atoms in benzene and the “covalent glue” holds more atoms together than just the vicinal (1,2) carbons.

As a second example, it is interesting to look at the ratio V_X_(1,3)/V_X_(1,2) in AB_n_ (n = 3 or 4) systems. This quantity compares the ligand-ligand (or B-B) interaction to the central atom-ligand interaction (or A-B). For methane, the ratio is 17/753 = 0.02 where the energies are quoted in kJ/mol. For BH_3_, BCl_3_, BF_3_ and BO_3_^−^ the ratios are respectively 63/373 = 0.17, 88/327 = 0.27, 92/276 = 0.33 and 121/293 = 0.41. The ligand-ligand interactions become increasingly more important in these systems to the point that they cannot be ignored in explaining the overall stability of the molecule. Indeed, methane is clearly held together by C-H interactions rather than H–H, with its piffling ratio of 0.02. In contrast, the well-studied BF_3_ molecule displays F-F interactions that are one third of the strength of the B-F interactions. Hence, it is tempting to update its Lewis diagram and perhaps draw dotted lines between the F atoms in the knowledge that the full stripes between B and each F atom do not suffice.

To reply specifically to the subquestions above, the BN interaction has a covalent strength (i.e. |V_X_(A,B)|) of 184 kJ/mol in BH_3_···NH_3_ while |V_X_(B,F|) = 276 kJ/mol and |V_X_(N,H)|) = 707 kJ/mol. Note that the level of theory is the same as above and that BF_3_···NH_3_ was not studied in that paper (ref.60). In terms of halogen-bonded systems an extensive IQA analysis can be found in ref. [172]. For example, in FBr···NH_3_, the absolute value of the exchange (i.e. covalent) energy between Br and N is 251 kJ/mol (optimised at MP2/aug-cc-pVTZ level). Unfortunately, this article did not study a hydrogen-bonded system in which (the electron acceptor) FBr is the same, as requested. Finally, a profile of V_X_(A,B) depending on the internuclear AB distance is rarely published but a very important example has already been discussed in Section 2.11 in connection with new BCPs emerging in the formation of water (ref [139].).

## Section 2.4

### Brinck and Borrfors ask:

The concept of exact boundaries and the non-overlapping nature of topical atoms is advantageous for several reasons as discussed in the article. However, it seems best suited for analyzing molecules in condensed phases where there are no voids between atoms. How are the boundaries of atoms determined in dilute gases, where the distance between atoms belonging to different molecules can be very long? Similarly, how does the concept work when analyzing the distance dependence of an intermolecular interaction? I realise that this subject is touched upon in Section 2.4, but it seems to us that the text has more questions than answers.

### Reply:

Indeed, a molecule in the condensed phase will be completely bounded by interatomic surfaces, which are topological objects. It is perspicacious to query the boundaries of molecules in a dilute gas. One may be tempted to take a given constant electron density contour as the outer boundary of a molecule. This is what Bader and co-workers did in the 1980s. Such a contour is, strictly speaking, not a topological object. One is forced to choose an electron density value for the contour, which is against the minimal parameter-free nature of QCT.

Figure 5 shows an example where methanal…chloroform would be alone in the Universe, as a free complex. This state is more extreme than the dilute gas situation. In fact, one can successfully argue that a free complex does not exist. In reality the Universe is full of molecules (even when far apart) and hence a totally free molecule is a figment of the imagination. Thus we only need to think about the dilute gas situation (in the context of the posed question). The answer to the boundary problem is that, actually, even a molecule in a dilute gas is bounded everywhere by interatomic surfaces. In other words, even if one has to go out tens of Å one will eventually come across a BCP (admittedly with very low electron density) acting as the centre of an interatomic surface. The latter will be featureless (i.e. no dents or nearly flat) but it will still be a topological boundary, formally.

## Section 2.5

### Mo, Danovich, and Shaik ask:

Force fields are built at the atomic, not electronic, level. As such, these have nothing to do with the spacing-filling nature of topological atoms. Unlike most force fields, reactive force fields (ReaxFFs) adopt a bond-order formalism in conjunction with polarisable charge descriptions to describe both reactive and non-reactive interactions between atoms. In other words, ReaxFFs do not divide bonded or non-bonded interactions as well (Sections 2.4 and 2.5). Can the author respond to these concerns?

### Reply:

Classical force fields do indeed miss electronic effects, which is why special correction terms are typically added ad hoc, such as an out-of-plane potential (as a quadratic function of an out-of-plane coordinate). In contrast to the statement above I do think that ReaxFF divides bonded and non-bonded interactions. A key paper [173] explains that ReaxFF divides the system energy up as follows:

$${E}_{system}={E}_{bond}+{E}_{over}+{E}_{under}+{E}_{val}+{E}_{pen}+{E}_{tors}+{E}_{conj}+{E}_{vdWaals}+{E}_{Coulomb}$$

It is clear that ReaxFF follows the architecture of non-reactive force fields by introducing the typical bonded/non-bonded dichotomy with for example E_val_ and E_tors_ covering for the bonded part, and E_vdWaals_ and E_Coulomb_ covering for the non-bonded part. However, the bonded/non-bonded imprint is deeper than just the recovery of these typical terms. Specifically to ReaxFF there are two extra energy terms (corrections actually), E_over_ and E_under_, which respectively correspond to an over-coordination and under-coordination penalty. These artificial terms result from holding on to the idea that a carbon must have a total bond order of maximum 4. This constraint is dictated by valence theory of bonding and would not appear in FFLUX. In FFLUX, atoms are surrounded by however many atoms are present and its machine learning operates on this environment without preconceived idea of connection or coordination. In summary, ReaxFF structure is deeply affected by a sharp distinction between bonded and non-bonded interactions, while FFLUX is not.

## Section 2.5

### Misquitta comments:

In Section 2.5 you wrote “…why bonded atoms do not interact electrostatically too…” This is a very good question. Of course they do, and such interactions are taken into account in FFs like AMOEBA. However, they also interact via all the other types of interaction: dispersion, polarisation, exchange. You ask about the dispersion in the next para: you are right, the dispersion should also occur between all atoms. This is indeed done in many models of the dispersion energy. Examples are any dispersion correction to DFT, MBD, AMOEBA,… pretty much any FF will include these interactions.

### Reply:

I am pleased that you find this a good question; it does not seem to be asked anywhere, let alone answered. Looking at a fairly recent paper explaining AMOEBA and its capabilities [174] the following functional form is given:

$${E}_{system}=\left({E}_{bond}+{E}_{angle}+{E}_{b\theta }+{E}_{oop}+{E}_{torsion}\right)+({E}_{vdWaals}+{E}_{elec}^{perm}+{E}_{elec}^{ind})$$

where the first brackets embrace the bonded terms and the second brackets the non-bonded terms. The 7^th^ term takes care of the electrostatic interactions but I cannot see how DMA multipole moments can converge for bonded atoms (i.e. in a 1,2 connectivity relationship). The matter is not discussed in the paper. I doubt that AMOEBA takes into account bonded electrostatic interactions or have I missed something (related to the induction energy)?

## Section 2.6

### Szalewicz asks:

The author refers in several places to a method of constructing FFs based on QCT and called FFLUX. This method is not described in the paper, so it is difficult to evaluate it. The author states that “FFLUX sidesteps perturbation theory”. Does it mean it is based on a supermolecular method? After all, as already mentioned, QCT is not a stand-alone method and has to use some QM approach for solving Schrödinger’s equation.

### Reply:

I am sorry that FFLUX has not been described: with more than 15,000 words the manuscript was already far above the expected length and its content aimed at focusing on non-covalent interactions rather than force field design. Still, ref. [77] describes QCTFF, which, as a precursor of FFLUX, covers many missing details. Yes, it means that FFLUX is based on the supermolecular method. QCT indeed uses a QM approach (any, in fact) for solving Schrödinger’s equation, nothing has ever been claimed to the contrary.

### Szalewicz comments:

The author asks “how robust is the definition of dispersion outside the polarisation approximation”. The answer is that the polarisation approximation, which is now often called Rayleigh-Schrödinger perturbation theory (RSPT), is based on the assumption that there are two (or more) interacting monomers, each of them a well-defined atom or molecule when in isolation. Thus, RSPT does not apply rigorously to long-range electron correlations within single large molecules. However, there are various approximate methods applying RSPT concepts to such systems. It is best to reserve the name dispersion energy to the case of two interacting subsystems. Coupled-clusters (CC) methods and other advanced electronic structure approaches, even many-body perturbation theory based on Møller-Plesset’s (MP) partition of the Hamiltonian, have no problem with describing long-range correlation within molecules, while DFT fails at it.

### Reply:

Thank you for this clarification.

### Szalewicz comments:

“A second problem with perturbation theory is that, at short-range, Rayleigh-Schrödinger perturbation theory actually breaks down because there is no unique definition of the order of a term in the perturbation expansion”. It appears to me that the definition of order in RSPT is completely unique. Moreover, RSPT does not break down, it just becomes unphysical (too negative) at close range since it neglects electron exchanges between interacting systems which lead to positive contributions.

### Reply:

“Unphysical” a matter of semantics. More precisely, I was referring to two problems actually, which arise if one constructs a perturbation theory that takes as its expansion functions wavefunctions that have been antisymmetrised over the whole complex AB,

$$\left|{\Psi }_{A}{\Psi }_{B}\rangle =\widehat{\mathrm{A}}\right|\left.{\Psi }_{A}\rangle \right|{\Psi }_{B}\rangle$$

where $\widehat{\mathrm{A}}$ is the antisymmetry operator acting on the electrons of both monomer *A* and *B*. Note that the product wavefunction $\left|{\Psi }_{A}\rangle \right|{\Psi }_{B}\rangle$ is antisymmetric with respect only to permutations of the *A* electrons among themselves and of the *B* electrons among themselves. Note also that these are the expansion functions of SAPT and hence the problems described here do not apply to it.

Now, one problem follows from the fact that the $\left|{\Psi }_{A}{\Psi }_{B}\rangle \right.$ wavefunctions are not orthogonal (whereas $\left|{\Psi }_{A}\rangle \right|{\Psi }_{B}\rangle$ are, even at short range). Thus, $\left|{\Psi }_{A}{\Psi }_{B}\rangle \right.$ cannot be eigenfunctions of an Hermitian operator because they must always be orthogonal. It then follows that we can no longer use standard RSPT, and there is no unique replacement theory within this *ansatz*. The second problem follows from a short derivation (omitted here) showing that a first-order quantity ends up equal to a zeroth-order quantity, thereby contradicting a basic assumption of perturbation theory.

### Szalewicz comments:

“Short-range perturbation theory is computationally expensive because one needs to take into account the other molecules that a given molecule interacts with". Yes, one has to take into account the other molecules, but if this were not needed, there would be no interaction. Furthermore, SAPT based on MP or CC description of monomers [SAPT(MP/CC)] is about as expensive as the MP/CC methods used, while SAPT based on DFT, SAPT(DFT), has only an order of magnitude worse scaling than DFT. Thus, SAPT is as expensive as it should be.

### Reply:

My statement was only made to support the choice of proceeding with supermolecular wavefunctions in the strategy behind FFLUX’s construction. The idea is that, if one is forced to consider the wavefunction of the interacting partner anyway, one may as well work with supermolecules and partition them according to QCT.

### Szalewicz comments:

Continuing the previous point: “In contrast, no such knowledge is required for long-range perturbation theory”. Actually, also in the asymptotic expansion, one has to take into account the other molecule. While this expansion uses monomer properties, the interaction energies are obtained from formulas involving both monomers. The use of monomer properties extends also to close-range SAPT. For example, the dispersion energy is an integral involving the density–density response functions of monomer *A* and of monomer *B*. Calculations of these functions is the major computational effort, while using them in a dispersion energy expression is much less time consuming.

### Reply:

Maybe my statement has been misunderstood. I simply stated that the wavefunctions of the two interacting monomers are cleanly separated, quantum mechanically. This treatment saves a lot of computation time and applies only to long-range (thus the comment on close-range SAPT is beside my point). One can calculate the multipole moments and distributed polarisabilities of one monomer without knowing with which other monomer it will interact. In other words, the electronic information (both field and response) of one monomer is independent of that of the other. This approximation is a huge computational advantage. It allows one to use simple analytical formulae (mentioned in the commentary above) to calculate the interaction energy (electrostatic, induction, dispersion) between two monomers.

### Szalewicz comments:

“As short-range perturbation theory is computationally so expensive, one may wonder why not use supermolecular wavefunctions?”. One reason is that, as already stated, SAPT(MP/CC) is about as expensive as the corresponding supermolecular variant of MP/CC. It also gives as accurate interaction energies as the supermolecular approach based on a given MP/CC variant (or more accurate: SAPT(HF/CCSD) is significantly more accurate than supermolecular HF/CCSD). Thus, for the cases where accuracy is similar, one can use either SAPT or supermolecular approach. The big difference is that the latter gives just the interaction energy as a single number, wheres SAPT interaction energy is composed of physical contributions. Clearly, SAPT is a better choice. The picture changes even more to SAPT advantage when we include SAPT(DFT). This method is about as accurate as CCSD(T), but orders of magnitude faster due to the O(N^5^) vs. O(N^7^) scaling. Therefore, as already mentioned, SAPT(DFT) provided the most accurate interaction energies for a set of large dimers, see ref.. [165]

### Reply:

Thank you for pointing out that SAPT(HF/CCSD) is more accurate than supermolecular HF/CCSD. However, at the current stage of development of FFLUX, IQA “undoes” the single number that a supermolecule leaves on with, thus SAPT is actually not needed, frankly. That SAPT(DFT) is about as accurate as CCSD(T) is also worth contemplating but perhaps more is needed to tip the balance away from supermolecules. First, if one wants to design a fully consistent and streamlined atomistic force field then an atomic partitioning of SAPT is needed. The two proposals A-SAPT and F-SAPT appear to be problematic. Second, the gain of two orders of magnitude is very welcome but only matters in the machine learning training part, not in the use of the trained models during molecular simulation. The latter is critical in the practical use of the force field whereas fast training is nice to have but not vital, due to its one-off nature (burdening only the force field creator).

### Misquitta comments:

The criticism of perturbation theory strikes me as unwarranted: it is perfectly possible to lay out the strengths of FFLUX and IQAs without criticism of perturbative methods. But not all of the criticism seems warranted. The rigid-body assumption of most force fields based on perturbation theory is valid, but just as FFLUX is based on rigid-body data and the parameterisation needed for handling flexible systems is learned, the same can be done here. Indeed, the AMOEBA FF is one of many modern FFs that are very much based on perturbation theory (in fact they use SAPT and SAPT(DFT)), multipoles (currently GDMA), polarisabilities, and also works for flexible systems. Of course there are assumptions made and some of these lead to a loss in accuracy.

### Reply:

I am sorry if the criticism came across as too harsh. For what it is worth, the tone originates from a perceived frustration that perturbation theory compelled molecules to be treated as rigid bodies for decades. Even today, research fields such as polymorphism prediction categorises flexible molecules as a more challenging class than rigid ones. In an ideal world, no such distinction is necessary, as is the long-term intention of FFLUX. The “conversation platform” that the current article (and subsequent discussion) is part of was taken as an opportunity to stir things up a little and hence some statements may have been “louder” than necessary.

Time will tell if the original design for FFLUX bears fruit. Its strength should come from its minimality (not simplicity!) and if true, there is no need “to make assumptions some of which lead to a loss in accuracy” as stated in the question above. Moreover, FFLUX is *not* based on rigid-body data, as wrongly presumed above. Instead, FFLUX is trained on flexible molecules according to a variety of geometric distortion methods, none of which could have been discussed in the main text due to space restriction. Originally [175] normal modes were used to systematically distort, from its equilibrium geometry, a given molecule that is trained for. Later, training geometries based [176] on the Protein Databank (PDB) and temperature-controlled MD trajectory-based distortions were introduced [177]. More ways are possible.

### Misquitta asks:

You have mentioned the use of QTAIM multipoles in evaluating the electrostatic energy in FFLUX. But what about polarisabilities and dispersion coefficients? Are these used in FFLUX? If I understood the method correctly, polarisabilities are not needed as the effects of polarisabilities are learned. But is there a dispersion model included in FFLUX, and if so, how is it used? There is certainly a correlation part of the IQA approach, but it is not clear how this is computed using DFT and how it is incorporated into the FFLUX model.

### Reply:

There are no polarisabilities in FFLUX as explained in Section 2.6. The viewpoint behind this decision is that FFLUX focuses on the result of the polarisation process rather than the response function governing it. This decision is partially motivated by the desire to open up the treatment of *intra*molecular polarisation. Its treatment is a “conceptual victim” of plain-vanilla perturbation theory, which is deeply tied in with *inter*molecular polarisation.

Maintaining and transferring this philosophy from polarisation to dispersion leads to an absence of dispersion coefficients C_n_ (n > 5). Inspired by the Casimir-Polder identity, those coefficients can be seen as the equivalent of the polarisabilities previously discussed. Logically FFLUX also avoids the use of dispersion coefficients and instead directly accesses the electron correlation energies made available by IQA. As such, any effects that dispersion causes, both at intramolecular and intermolecular level is captured, even at intra-atomic level. The effectiveness of this approach is illustrated by our study [163] on water clusters showing that (i) most of the cohesion in the water clusters provided by electron correlation comes from intramolecular energy stabilisation, (ii) hydrogen bond-related interactions tend to largely cancel each other, and (iii) electron correlation energies are transferable in almost all instances within 1 kcal mol^−1^. Moreover, we already know [82] that atomic electron correlation can be successfully machine-learnt, and with few training geometries as a bonus. Thus an avenue, novel compared to other force fields, is open to full implementation for use in molecular dynamics simulations.

### Misquitta comments:

(1) It is indeed true that methods like SAPT are designed to work with rigid fragments, but this is true of any quantum method that treats nuclei as classical.

(2) SAPT potentials for flexible systems have been computed (water, HF…) These are a few, but it is possible to do it.

### Reply:

(1) I believe that this is incorrect: FFLUX (currently) treats nuclei as classical but it can handle flexible fragments. Moreover, the fact that SAPT was designed to work with rigid fragments was not caused by treating nuclei as classical.

(2) That is good to know. However, with FFLUX, flexibility is the norm and its flexible systems are thereby growing each year, far beyond a few.

### Misquitta comments:

You make a valid point about the correlation between the ends of a long chain molecule. While methods like SAPT are not naturally suited to handle this, the F-SAPT method from the Sherrill group can compute such interactions and is available in the Psi4 code.

### Reply:

Thank you for pointing this out. The main text now reflects this point. I agree that SAPT is not naturally suited to handle this.

## Section 2.7

### Szalewicz comments:

“A damping function is a mathematical function that prevents an energy from becoming unreasonably large (and even infinite) at short range. Put bluntly, a damping function is an artificial device that cleans up a problem created by failing physics.” A damping function is actually a very physical concept. The asymptotic expansion of interaction energy is obtained by using the multipole expansion of the interaction potential which neglects several terms in the so-called bipolar expansion. The multipole expansion is correct only at very large separations and is only asymptotically convergent. However, the neglected terms in the bipolar expansion can be accounted for [61, 178, 179]. Their effect can be partly expressed as damping functions.

### Reply:

**Fig. 9:**
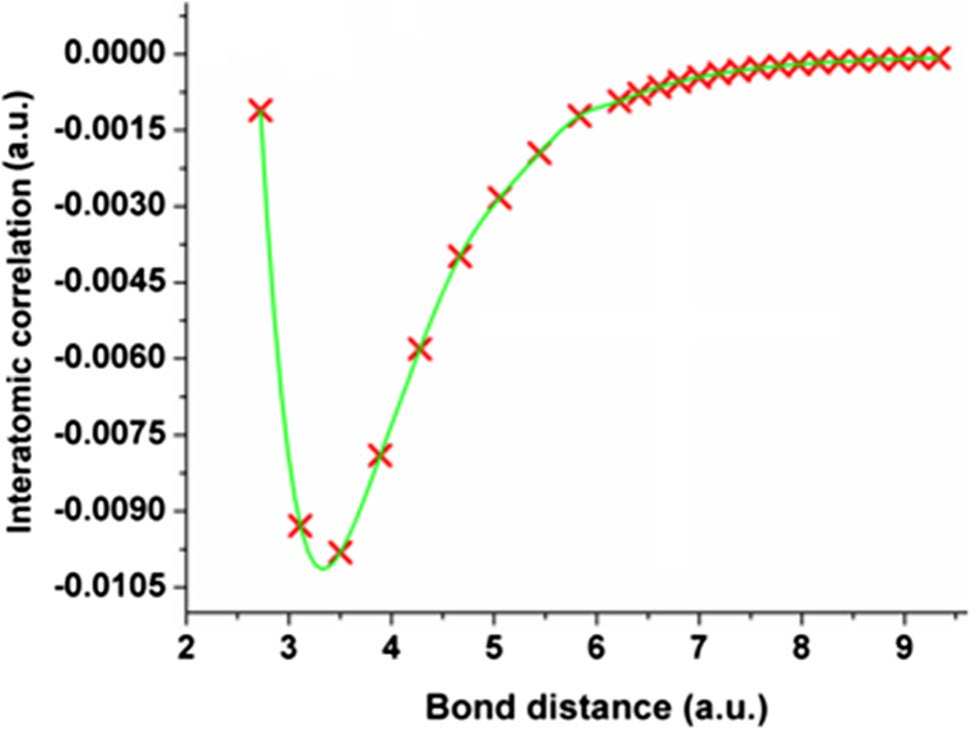
Interatomic correlation energy ${V}_{ee,c}^{AB}$ as a function of internuclear distance for the argon dimer

I do not think that a damping function is physical and want to use electron correlation as an example of an interaction to explain why. Equation (5) of the main text defines ${V}_{ee,c}^{AB}$, which is the interatomic correlation energy. Figure 9 plots this value for the argon dimer.

Between about 5 and 9 a.u., ${V}_{ee,c}^{AB}$ can be accurately fitted with an inverse sixth power of r_AB_. If one wants to use the resulting equation at short range then one heads for a singularity at r_AB_ = 0. To avoid this a damping function is typically invoked. In other words, such a function corrects for the unfortunate decision of projecting long-range behaviour onto a short-range regime. However, the ${V}_{ee,c}^{AB}$ profile captures the physics of the short-range regime, while the extrapolation of the inverse sixth power law unto this regime, does not. In this sense, a damping function is mathematical device. Following the benchmark paper [94] by Tang and Toennies, a (universal) damping function f_2n_(R) is an incomplete gamma function. It appears that no physical (i.e. theoretical, quantum mechanical) derivation for this function is given although the parameter *b* that it contains is said to have a physical meaning.

### Szalewicz comments:

Damping is indeed related to penetration of wavefunctions. This fact is, however, not “the (conceptual) picture of electron clouds” but a hard fact resulting from quantum mechanics. QCT violates QM by removing the overlaps of wavefunctions.

### Reply:

I do not think that quantum mechanics itself sees the wavefunction of the hydrogen molecule (to take a prototype example) as a system of two overlapping hydrogen atoms. Yes, this is a popular way to attack the problem of solving the Schrödinger equation of H_2_ but not the only way. For example, inspired by work of G.G. Hall, Mura and Handy developed [180] non-overlapping (cuboidal) basis functions. This shows that overlap is not a hard fact of quantum mechanics but a consequence of the choice to construct multi-electron wavefunctions from infinitely extending one-electron wavefunctions/orbitals.

Secondly, QCT does not violate quantum mechanics. Firstly, because overlap is not intrinsic to quantum mechanics and secondly because even if it was QCT does draw from overlap matrices to define various quantities [181]. For example, the exchange energy between tow topological atoms *A* and *B* can be defined as

$${V}_{x}\left(A,B\right)=-2\underset{{\Omega }_{A}}{\overset{}{\int }}{d\mathbf{r}}_{1}\underset{{\Omega }_{B}}{\overset{}{\int }}{d\mathbf{r}}_{2}\sum_{i}\sum_{j}\frac{{S}_{ij}({\mathbf{r}}_{1}){S}_{ij}({\mathbf{r}}_{2})}{|\mathbf{R}+{\mathbf{r}}_{2}-{\mathbf{r}}_{1}|}$$

where the denominator is the distance between two infinitesimal portions of electron density, one in the atomic volume ${\Omega }_{A}$ and the other in ${\Omega }_{B}$, and ${S}_{ij}$ is a convenient overlap function defined in terms of molecular orbital ${\Psi }_{i}$ by ${S}_{ij}\left(\mathbf{r}\right)={\Psi }_{i}\left(\mathbf{r}\right){\Psi }_{j}\left(\mathbf{r}\right)$.

### Misquitta asks:

There is much made of the absence of a penetration term in the QTAIM/IQA approach. This is of course technically correct. The QTAIM domains do not inter-penetrate and so there is no “penetration” energy. But consider the usage scenario that is described in 2.7 and 2.9. The electrostatic energy between near atoms (< 7 Å separation, but if I understood earlier papers correctly, this is probably < 4 Å if quadrupole moments are included on all atoms) is computed using the QTAIM densities as a 6-dimensional integral, and for atom pairs separated by more, the multipole expansion is used, truncated at a specified rank.

Now compare this with a similar approach for approaches using inter-penetrating AIMs such as the Hirshfeld, H-I, MBIS, ISA, GEM and other methods. Here we have two options: either do exactly as above, i.e., use the exact expressions for nearby atoms which have inter-penetrating densities, and the multipole expansion for the rest, or we could alternatively use "damping functions" like those derived by Slipchenko and Gordon to recover the missing penetration energy.

An aside here: as the divergence of the electrostatic expansion is manifested at separations high up on the repulsive wall for most (neutral) complexes when multipoles are limited to rank 4 or less, it is quite possible to simply ignore the divergence, and include the missing penetration energy via a separate term. I use this approach but consider it less satisfying that the other mentioned previously.

How are these two approaches different in practice? They are not. Both methods need to compute the near-atom electrostatic energy using the exact approach, and both use the multipoles only for the far atoms. Details do matter here and it is quite possible that one approach may need a smaller near-atom domain, but otherwise they are the same in practice.

If I had to choose (and I am certainly not unbiased) I’d choose the latter approach as even if I had to make a model for the penetration, I know the analytic form for such a model, and even if I had to compute near-near electrostatic energies numerically, I could do this using basis expansions for the inter-penetrating atomic domains, as is done in the GEM model. These strategies do not seem available to FFLUX. Could you comment on these issues?

### Reply:

The section was kept brief in order to highlight its central idea without dilution and because of the already ample length of the whole article. Moreover, the full impact of the no-penetration idea is still being worked out in our research. Let me first comment on the first point raised. Yes, we use the 6D integration to calculate the electrostatic energy between two atoms when they are close to each other (in order to avoid multipolar divergence). The distances mentioned in the question are not accurately quoted but the full story is in our extensive paper [117] on crambin where detailed convergence distances (“radii”) are given. The actual values of these distances is not important in the current discussion (but the convergence distances are more favourable than the 4 Å and 7 Å mentioned in the question).

I interpret the first point raised as a challenge to the relevance of non-penetration if the short-range interatomic energy is calculated at without multipolar expansion anyhow. If correct, then this point is valid. Let us convey the main message of Section 2.7 better and discuss the ultimate goal of FFLUX, which is incorporating transferability into its design. FFLUX will carve out an atom from a *small* condensed matter environment, train for its properties and then predict them for that same atom sat in a *large* condensed matter environment. The bottomline is that this atom is like a piece of a jigsaw, finite and non-overlapping. As such, it is readily quantified and easily controlled in terms of its electrostatic contribution (whether energy of potential). In that context a trained FFLUX atom can just be slotted in without having to worry about damping functions, that is all. The only error made is that due to inevitably imperfect transferability.

However, the methodology described above does not yet exist although software has been prepared to attain a proof-of-concept. Molecular dynamics FFLUX simulations currently occur at what we call the “monomeric level” but plans exist for “oligomeric simulations”. In the practical regime of monomeric modelling, monomeric wavefunctions do overlap, which is against the QCT philosophy. Currently we ignore any penetration effects but they, and other effects, have been quantified in unpublished work comparing a water dimer wavefunction with a superposition of two monomeric wavefunctions of water. We may well have a look at the work mentioned by the questioner in order to improve QCT monomeric modelling. However, it should be emphasised (again) that monomeric simulations are only meant to be transitory and thus a serious research investment into damping functions is not planned.

## Section 2.8

### Szalewicz comments:

“There is considerable evidence that multipolar electrostatics overcome the limitations of the ubiquitous point-charge approach. In particular, a model of one point-charge for each atom fails to capture the anisotropic nature of electronic features such as lone pairs or π-systems." Indeed, the multipolar electrostatics is an important concept. However, the point-charge approach works sufficiently well for most current applications. Our very accurate potential energy surfaces for water, [97, 182–184] a system with lone pairs, include charged off-atomic sites that provide a truthful representation of the electrostatic energy. Our potential energy surface for the benzene dimer [185] describes π—π interactions so well that it for the first time identified the true global minimum of this dimer. For molecules with tens of atoms, off-atomic sites are not needed anymore as the partial charges on atoms provide a sufficient account of the anisotropy of the electrostatic energy, see for example ref. [186].

### Reply:

In my statement above I explicitly wrote “one point-charge for each atom”, which the commentator seems to ignore. The mentioned water systems all contain point-charges on off-atomic sites, which compensate for the shortcomings of the one-nucleus-one-point-charge model. The mentioned benzene dimer was modelled by 13 additional off-atomic sites and this again does not serve as a counterexample for the superiority of multipolar electrostatics. Finally, the 21-atom system of ref., [186] or the cyclotrimethylene trinitramine (RDX) molecule, was curiously represented by only 13 charges, which were non-linearly fitted to asymptotic electrostatic energies. Presumably, “asymptotic” means long-range, in which case a point-charge becomes an exact representation (see ref., [76] for example). If so, one cannot draw the above conclusion about anisotropy being well accounted for.

### Mo, Danovich, and Shaik ask:

The author advocates their newly developed machine-learning-based force field FFLUX, which is characteristic of the multipolar expansion (in comparison with the point-charge approximation in current force fields) and (hopefully) can solve the problems the author raised. The author discusses the drawbacks and failures of currently popular force fields like Amber, CHARMM, GROMOS and OPLS. Is it possible to show readers results of the new FFLUX force field for head-to-head comparisons with the criticised force fields?

### Reply:

The criticism stems from papers (written by authors other than ourselves) that show that the predictive power of classic force fields is unfortunately poor (see ref [105] for an example). Head-to-head comparisons between FFLUX and classical force fields do not yet exist but will take place in the future. However, some time ago we have performed molecular dynamics simulations on liquid water with QTAIM multipolar electrostatics, albeit on rigid waters. Note that FFLUX enables flexible molecules (see ref [85]) and that capability has not been test via comparisons with classical force fields. However, our rigid-body molecular dynamics work (e.g. ref [187]) made comparisons with the TIP4P, TIP5P and SPC/E potentials. For example, whereas these force fields respectively put the maximum density of water at − 15 °C, + 4 °C and − 38 °C, we predicted (using the molecular dynamics program DL_MULTI) a temperature of + 6 °C while experiment says + 4 °C. Considering that TIP5P was specifically designed (by fitting the potential against experiment) to correctly predict the temperature of maximum density, we were doing very well with our first principles approach. Furthermore, other thermodynamic properties can well deviate from experiment by a previously seen 20–40% but our calculations were never more than 100% off. Some force fields yield such large (and even larger) deviations for one property while sometimes being spot on for another property.

Finally, some time ago, we compared QTAIM multipolar electrostatics (without machine learning, thus rigid only) against four popular point-charge models (TAFF, OPLS-AA, MMFF94x and PFROSST) for a hydrated serine [188]. We concluded that, at static level, multipolar electrostatics best reproduces the ab initio reference geometry. Secondly, at dynamic level, multipolar electrostatics generates more structure than point-charge electrostatics does, over the whole range.

### Mo, Danovich, and Shaik ask:

The author claimed that “an atomic charge is a measure or product of charge transfer”. But charge transfer could occur in two directions, and the “net” transfer may underestimate the energy benefits. Besides, polarisation too could lead to atomic charges in population analyses. How does the author resolve these difficulties?

### Reply:

There seem to be two points in this questions: (i) net, overall charge transfer *versus* “resonance snapshot” charge transfer, and (ii) the (different) roles of charge transfer and polarisation.

On the first point, QCT allows one to analyse “snapshots” of the electron distribution via the so-called electron distribution function, which has recently been reviewed [189] since its inception in 2007. This is not what we do here and thus we do not have access to potentially opposing directions of charge transfer. So, in principle the “difficulty” that the questioners mention above can be tackled, mindful however of the low probability (and hence relevance) of certain resonance structures.

On the second point, in one of my questions to your own article I have explained in detail the difference between charge transfer and polarisation according to QTAIM. Let me just highlight the essence here in order to keep the current article self-contained. Dipolar polarisation is defined by the intra-atomic dipole moment ${\varvec{\upmu}}\left(\Omega \right)={\mathbf{M}}_{1}\left(\Omega \right)=-\underset{\Omega }{\overset{}{\int }}d\mathbf{r}{\mathbf{r}}_{\Omega }\rho \left(\mathbf{r}\right)$, and only describes how the electron density is shifted away from the nucleus. On the other hand, “monopolar polarisation” is defined as $N\left(\Omega \right)=-{M}_{0}\left(\Omega \right)=\underset{0}{\overset{}{\int }}d\mathbf{r}\rho \left(\mathbf{r}\right)$, which essentially coincides with charge transfer (e.g. from free atoms to the same atoms in a system). Hence, the last statement of the above question cannot be supported.

## Section 2.9

### Szalewicz asks:

The QCT O–H contribution to the electrostatic energy of the water dimer at the van der Waals minimum is − 415 kJ/mol (Fig. 6). However, the total electrostatic energy at such configuration is about − 30 kJ/mol. Thus, atom–atom contributions are an order of magnitude larger in absolute value than their sum. Does it not appear a bit unphysical?

### Reply:

No, I believe this value is physical and seems right in the context of Table 1. This table gives the somewhat different value $({V}_{elec}^{HB})$ of − 442.0 kJ/mol (6% difference due to a different level of theory), which provides a sense of how large intra-atomic and interatomic energies add up to energies that are typically an order of magnitude smaller. This phenomenon has been seen within a molecule too and shows how chemistry emerges as a science of “atomic cancellation”, both at molecular and supermolecular level. Philosophically, one can put this phenomenon in a wider context where interaction energies becomes successively smaller, starting with the formation of an atomic nucleus (from protons and neutrons), over the formation of an atom (from a nucleus and electrons), and the formation of a molecule (from atoms) to the formation of complexes (from molecules).

### Szalewicz comments:

The paper states that QCT can provide means for constructing force fields (FF). However, QCT atoms in addition to several advantageous features, have one significant drawback from the FFs point of view: multipolar expansions based on QCT atoms converge very slowly, as seen in Fig. 6. The same problems were shown in ref [87] for distributed polarisabilities, where high-rank polarisabilities had to be included. In contrast, other distributed approaches [190–192] work well with up to rank 2 distributed polarisabilities.

### Reply:

Let us distinguish between multipole moments and polarisabilities, and discuss them one at a time. Firstly, in the work [87] of Ángyán et al*.* the main message is that QTAIM distributed polarisabilities are remarkably stable with respect to basis set extension. In contrast, Le Sueur and Stone had shown a year earlier that the Hilbert-space partitioning scheme leads to unphysically large distributed polarisabilities. By the way, we benefited from QTAIM’s stability, and thus practicality, while studying the polarisability of the water dimer (see our work [86] mentioned in the main article). As far as I can see, the work of Ángyán et al*.* does not discuss any convergence (of which expansion?) of distributed polarisabilities. Moreover, the highest rank values mentioned in that paper are dipole–dipole, which is not considered high rank.

Secondly, when it comes to multipole moments, the literature unfortunately keeps repeating the rather naïve and underdocumented opinion on poor convergence of the multipole moment of QCT atoms. This widespread opinion is probably caused by the statement in Stone’s book, which is based on a single and dated study. We took this convergence concern on board some time ago and carried out a lot of work to show that matters are more nuanced and not as bad as people think. We have shown before [109] that the electrostatic potential of a topological atom converges reasonably well in spite of its finite and typically cusped shape. This benign behaviour is due to the decay of the electron density inside the atom; if replaced by an artificial uniform density, the convergence is indeed very slow. Later work [193] directly compared the multipolar convergence of QTAIM moments with DMA moments. This work introduces a shift of the respective multipole moments to off-nuclear sites, which substantially improves the convergence of QTAIM for small van der Waals complexes and without changing the nature of the topological partitioning. For the larger test systems (DNA base pairs), QTAIM *surprisingly* already converges as well as DMA, without extra sites. Finally, our extensive study [117] on the small protein crambin shows that the multipolar electrostatics with QCT atoms is adequate with practical convergence radii.

### Misquitta asks:

The study on crambin that you refer to is a very nice one and is the kind of study we all need to conduct. I’d like to point out that Rob et al*.* [194] have done something analogous. I do have a critical comment that perhaps should be answered. If we read this paragraph, especially the very good comments made in the second half, together with Section 2.7 on the absence of penetration in this approach, then I see something to be concerned about. I have mentioned it above in my general comments, but it is worth re-stating some of these here. If the QTAIM moments can be used only after 7 Å, and the exact expression needs to be used at shorter separations, then does it really matter that there is no penetration in the QTAIM approach? As I stated above, even in a method with penetration, exactly the same needs to be done. So what is the difference?

### Reply:

Thank you for the kind words on the crambin study. However, I must point out again that the QTAIM moments can be used for distances well below 7 Å. Table 6 in that paper is a key table listing the smallest internuclear distances at which the multipolar expansion still converges, for all 15 possible element-element electrostatic interactions. For example, for H…H the distance is 2.1 Å while for S…S it is 3.7 Å. The difference, as asked above, is one of principle: in a pure QTAIM context one never has to worry about penetration. However, a method that does have penetration always leaves the user with the question: how large is the associated energy? Can it be ignored?

## Section 2.10

### Szalewicz comments:

The polarisation model criticised in Section 2.10 works very well in the first-principles potential energy surfaces developed by our group, see in particular ref.. [195] The polarisation catastrophe can be easily avoided by using a damped model. Indeed, in molecular dynamics calculations the use of such a model increases the costs significantly (nevertheless, polarisable FFs are now becoming the mainstream in biomolecular simulations). However, since this model introduces important physics into FFs, it is definitely worth the costs.

### Reply:

The intention is for FFLUX to avoid the polarisation catastrophe. Yes, polarisable FFs are becoming mainstream and FFLUX intends to join that effort, machine leaning of oligomers allowing, by capturing polarisation effects directly, by the already polarised multipole moments rather than by machine learning polarisabilities.

### Misquitta asks:

What do you mean by “perturbation theory…handles charge transfer in a ‘bolt-on’ manner…and… causes the polarisation catastrophe”? Long-range perturbation theory is valid only at separations for which orbital overlap is negligible. At such separations the charge transfer energy is zero and there can be no polarisation catastrophe. At any shorter separation we use a symmetry-adapted method like SAPT. This has exactly the same long-range form as Rayleigh Schrödinger Perturbation Theory but has none of the issues mentioned in this paragraph. Charge transfer is accounted for naturally and there is no polarisation catastrophe.

Classical polarisation models can be constructed based on these SAPT energies and these can be damped to remove the polarisation catastrophe. It is true that such models cannot describe the charge transfer energy but it is possible that models that allow charge movement (such as those from Jensen and other groups) will be able to capture the charge transfer energy too.

### Reply:

The original text has been slightly modified to shield against the comments above, which are reproduced here in full, in order to give further clarity. “Bolt on” means ad hoc and not streamlined.

### Misquitta comments and asks:

Later on in this paragraph you state that the multipole moments need to be computed on-the-fly in an iterative manner. Surely this is the correct physical process? As the geometry changes, so do the induced moments (and charge movement too). Any method attempting to describe the condensed phase or clusters will need to include these phenomena. It could be done through the physics of the polarisation model, or by developing a machine-learned model (FFLUX?) which is parameterised based on a *lot* of data. But this is a very real phenomenon.

### Reply:

Let us first be clear that the on-the-fly calculation does not apply to FFLUX. Returning to the main point of a polarisation process, sure, this is a real phenomenon but only of interest if one is specifically monitoring that process. It is then that a polarisation energy (proportional to the polarisability and the electric field’s magnitude squared) needs to be added. In the FFLUX framework this process is not explicitly monitored. Instead, FFLUX has been trained on a sizeable set of wavefunctions, one for each system geometry. The wavefunctions are obtained after self-consistent-field (SCF) iteration by some approximate method that solves the Schrödinger equation. For each previously unseen geometry, FFLUX predicts the properties of an atom of interest. The machine learning method does this essentially by interpolating (in a the high-dimensional space of internal coordinates) between the given training energies (and geometries). At no point does the machine learning monitor a polarisation process; it does not consider one electron density being induced into another. Hence, it does not see a polarisation process and thus it does not add a polarisation energy. If one insists on the latter, then it must already be included in the SCF energy determination of each training wavefunction. So I disagree that the phenomenon of polarisation needs to be included in all methods that describe condensed matter. FFLUX is such a method and it does not.

## Section 2.11

### Mo, Danovich, and Shaik ask:

Subsequently, the author deals with down-to-earth treatments of non-covalent interactions. QTAIM is one of the major tools in current analyses, and the descriptors such as bond critical point (BCP). Recently it has been argued that BCP does not correspond to a chemical bond. What is the author’s position on this issue?

### Reply:

The full meaning of a BCP has been precarious for a few decades but less-than-5-year-old studies (e.g. refs [138, 141]) have been stinging and increasingly worrisome. Yet, a 2021 study, [196] for example, shows awareness of the severe criticism on the real meaning of the BCP, yet confidently proposes a criterion using properties evaluated at the BCP. This study covers (via prototypical systems) an impressive range of non-covalent bond types. Looking beyond this paper, perhaps one can be left with the notion that useful chemical information can be extracted from the region near a BCP, even if it is not present.

Overall, I am concerned that a BCP may not work for the right reasons and advocate yet more research on the topic but without cross-purposes communication, and “put all of one’s cards on the table”, good and bad. Furthermore, I am reassured that bonding insight drawn from IQA is not affected by the ongoing BCP controversy. The least we can do for now is call a BCP by the safer, unloaded name of line critical point (LCP).

### Brinck and Borrfors ask:

There is a relatively long section on the BCP, but there is no strong conclusion on the relevance of the BCP. Does the BCP still serve a purpose, or has it been superseded by NCI and REG?

### Reply:

My position on the BCP issue has already been made clear in a reply to the previous question by Mo, Danovich, and Shaik. I believe that REG, when combined with IQA, produces valuable answers while addressing the same type of question that a BCP aims to address: where are the bonds in a system and what is their nature? REG can be used to answer this question, while not depending on (the vagaries of) BCPs. However, REG can answer more general questions than this one. Moreover, REG has the capacity to eventually supersede NCI because it is a more general and powerful idea that operates on valuable data such as IQA energies.

The proposal of the NCI method was inspired on the issue surrounding BCPs, or at least, is often invoked to remedy it. Looking at the practical use of NCI, the only quantity that matters is |$\nabla\uprho$|, never mind the reduced density gradient *s*, which is presented as a fundamental dimensionless quantity in density functional theory. This gradient is a ratio containing |$\nabla\uprho$| in its numerator. NCI’s “solution” to the sudden appearance or disappearance of BCPs is to inspect a 3D plot of |$\nabla\uprho$| and pick an *arbitrary* contour surface. If a BCP does not form (i.e. there is no very near zero-contour surface) then there are always other contour surfaces to plot. As such, NCI actually avoids topology and reverts back to simple contour plotting. Secondly, and more worryingly, the information about the extent and nature of the intermolecular interaction that NCI claims to offer is questionable in my opinion; it merely depends on an over-interpretation of λ_2_, which curiously leads to a repulsive interaction right in the middle of a system as stable as a benzene ring. Such a conclusion is incompatible with the physically meaningful energies offered by IQA and the corresponding insight (see the first example of the first question of Brinck and Borrfors).

## Section 2.12

### Mo, Danovich, and Shaik ask:

In Section 2.12, the author criticises the secondary electrostatic interaction (SEI) hypothesis by Jorgensen and Pranata. But the SEI concept can be easily understood and is thus welcomed by experimentalists although later computational studies showed that other factors notably σ induction and π resonance also contribute to the intermolecular bonding. There is no perfect interpretation so far. But the comparison with the football game is problematic as the scoring results from the cooperation within the team (such as field effects) rather than only the player alone. Can the author explain clearly his opinion that the Relative Energy Gradient (REG) approach can address his listed questions with easily understood solutions?

### Reply:

“Easily understood” does not mean correct. Ultimately, experimentalists need predictive tools that work and they themselves publish exceptions to the SEI, which means they feel SEI cannot always be trusted. So, maybe they already surmise reality is more complicated and a more sophisticated rule is necessary.

Perhaps the football metaphor has been misunderstood. Yes, there are normally 22 players that all do various things leading up to a goal but this is not how goals are explained (by professional commentators or the public). Equally, a chemist will not explain the anomeric effect by stating that there are 24 atoms in a given molecule, all moving around in various ways. Instead, the challenge is to tease out the most dramatic change that leads to a goal that is, the chemical phenomenon under study. This is what REG does, by ranking the slopes of the various energy contributions. The largest slope is then the contribution that “pushed hardest” for the overall phenomenon (i.e. the goal, the anomeric effect) to occur. If this slope is much larger than any other then the phenomenon is indeed largely due to one energy contribution (“one player”) but otherwise it could be due to a number of high ranking energy contributions.

## Section 2.13

### Szalewicz asks:

Figure 8 shows atom–atom correlation energies for a configuration of the water trimer. Some intermolecular atom–atom energies are of the order of − 200 kJ/mol. This is an even more pronounced problem than with Fig. 6 since the majority of intermolecular interaction energy for the water dimer is given by the Hartree–Fock (HF) level of theory and the correlation contribution is only of the order of − 10 kJ/mol.

### Reply:

This question is similar to the previous one. Figure 8 shows that these correlation energies can be as small as 2.5 kJ/mol (for H…H in water) or even as large as − 1658 kJ/mol, internally for an oxygen. Their magnitude is determined by the physics of electron–electron interaction and its mathematically minimal partitioning. If the energies turn out to be large then they typically participate in a “cancellation scheme” such as the remarkable near-cancellation of electron correlation energies between the negative H…X value and the positive X’…X values in [X’-H…X] systems mentioned in Section 2.13. In summary, it is not clear why large individual atom–atom energies would be a problem.

### Brinck and Borrfors ask:

Could you elaborate on the computational complexity involved in determining force field parameters for a new molecule in FFLUX? Is it sufficient to do a quantum chemical calculation on the molecule itself or is supermolecular calculations involving different combinations of molecules also needed? Is there a transferability of parameters between different systems? What are the main advantages of FFLUX in comparison with traditional force-fields? In simple terms, why do we need FFLUX?

### Reply:

There are several questions in one. I will start with the last question and then answer the others in reverse order.

We need FFLUX because of the accumulating literature evidence that standard force fields, even with all their versions and modifications, are not able to give a consistent and reliable answer on the structure and dynamics of oligopeptides, let alone proteins. My article briefly introduces the disconcerting study of Rauscher et al*.* [105] but there is a growing number of similar studies [197–199]. Many years ago I thought that it is better to overhaul the existing force field architecture and start afresh, with new principles. This ongoing effort has culminated into FFLUX, which is still under construction, given the *tabula rasa* at its origin.

At a technical and conceptual level FFLUX benefits from the listed advantages of QCT. More broadly, its design is “cleaner” (“more Occam”) than that of a classical force field. This may be seen by some as a merely philosophical plus but it already pays off by guaranteeing that electrostatics means electrostatics, for example. In other words, this energy component is very well-defined and not contaminated by any other contributions. As a result, it can be improved by itself without harming the overall energy prediction. In classical force fields such modularity does not take place alas.

Transferability is something that exists at the level of quantum chemistry, independently of machine learning. This point is often misunderstood by newcomers to force field design by machine learning. One of the reasons we use QTAIM partitioning is because of its high transferability. It is important to realise that FFLUX’s machine learning operates on already partitioned (i.e. atomic) quantities. Those newcomers often see this as a disadvantage, leaving the partitioning (and thus “transferability”) to the machine learning method itself. I disagree because, within a physics-based force field, it is safer to machine learn already partitioned quantities. For example, Behler’s deep neural net method partitions a system into atomic quantities “on the spot”, that is, during the training. This is a potential vulnerability because the neural nets are not guided by any physics during their partitioning. Finally, it should be mentioned that we have looked for transferability in the kriging hyperparameters. Although some weak “signals” have been observed, the matter is not closed and warrants further research with the latest stable version of our ICHOR/FEREBUS package.

Currently we have mostly published examples of force fields (i.e. kriging models) for single molecules. We call this the “monomeric level” and it enables one to carry out MD simulations already. We have tested the program DL_FFLUX (an in-house derivative of DL_POLY) on multi-nanosecond runs on thousands of atoms. Of course we can also perform geometry optimisations on single molecules. The next step is the construction of supermolecular complexes in a systematic way with the latest version of our in-house software ICHOR/FEREBUS. An early paper [200] delivered proof of concept and further promising preliminary results have recently been accumulated.

Finally, the opening subquestion is quite vague but it is perhaps helpful to simply highlight the challenge of *configurational flexibility* during training, as a main aspect of computational complexity. Even a simple system such as a water dimer poses difficulties in being efficiently captured in all its appearances in liquid water. How to construct a compact but well-informed sample pool is an ongoing topic of research.

### Mo, Danovich, and Shaik ask:

From the example discussed by the author, the REG analysis is sensitive to the computational level. For example, at the MP2/6-31G** level, oxygen atomic energy decreases by 8 kJ/mol by accepting one hydrogen (forming hydrogen bond). But CCSD value changes to 25 kJ/mol. Isn’t this disparity of concern to the author?

### Reply:

Yes, I am concerned about this but the discrepancy can be explained and the way forward determined. In short, CCSD is the ultimate way to go because it is more realistic than MP2. In fact, any coupled cluster wavefunction (e.g. CCSD(T)) is, compared to any Møller-Plesset wavefunction (e.g. MP2, MP3, MP4SDQ). To understand why, we remind ourselves that the atomic volumes, used to obtain the correlation energies, originate from the one-electron density. For MPn, this electron density is the Hartree–Fock density. The latter is employed because MPn correlation only affects electron–electron terms. However, for CCSD, an electron density is employed that now includes correlation. We have observed in ref [162] that CCSD picks up more subtle effects than MP4SDQ. In summary, CCSD is the way forward but I wish that MPn would numerically be closer to it.

### Misquitta asks:

What is the overall accuracy of the FFLUX method and how do you expect it to compare with conventional Quantum Chemistry techniques like CCSD(T)? Perhaps this is not a relevant question; if so, how should we gauge the accuracy of FFLUX?

### Reply:

First it should be clarified that FFLUX predictions (as well as IQA energies) are as accurate as the accuracy of the wavefunctions that feed FFLUX. Whereas IQA has “operated” on Hartree–Fock, CASSCF and CI wavefunctions since inception, [44] only relatively recently it has been made compatible with DFT, [50] MPn [80] (n = 2, 3 or 4) and CCSD, [162, 201] CISD and CCSD(T) [201]. In FFLUX there are two types of errors due to (ii) 3D or 6D integration over the atomic volume(s), and (ii) the predictive error of the machine learning method. Let us discuss them in turn.

The first type of error is due to the algorithmic complexity of the multidimensional integration over the complicated shapes that topological atoms may display. While a sizeable quadrature grid can reduce the error to near noise (~ 0.1 kJmol^−1^ or less), computational expense may return [160] recovery errors for the (electron) correlation energy of about 0.6 kJmol^−1^ for a modest number of grid points. A recovery error is the difference between the original system energy and that obtained from the sum of atomic contributions. The topological partitioning suffers less from the challenge of atomic integration than it used to thanks to better algorithms and computer hardware. Yet, it remains a disadvantage compared to other partitioning methods (especially in Hilbert space), whose rather trivial partitioning complexity barely demand computer resource.

The second type of error has not been discussed because it perhaps ventures too much into the area of machine learning and is thus deviates from the main topic of non-covalent interactions. This error is constantly being updated as our machine learning continues to improve but an early study [82] showed that Gaussian Process Regression (aka Kriging) can predict the total correlation energies for all test geometries of the water dimer (amongst others, and at MP2/uncon-6–31 +  + G(d,p) level of theory) to within 0.5 kJmol^−1^ of the original (exact) training energies. Looking at the electrostatic energies (only for atoms in a 1,4 relationship or higher) for all 20 natural amino acids reveals [202] that (for 200 unseen test geometries for each amino acid) all amino acids have a mean prediction error below 5.3 kJmol^−1^, while the lowest error observed is 2.8 kJmol^−1^. The mean error across the entire set is only 4.2 kJmol^−1^ (or 1 kcalmol^−1^). While respectable for say molecular dynamics simulations, polymorphism modelling demands errors of the order of 1 kJmol^−1^ or less. Promising unpublished results shows that our current research follows this direction of travel.

### Misquitta asks:

How are many-body effects handled in FFLUX? Are they learned, and if so, how well is the model able to pick up many-body non-additive polarisation and dispersion effects?

### Reply:

Indeed, many-body effects are learnt. Even simple molecules such as methanol [177] already exhibit subtle interactions such as the oxygen’s lone pair interacting with the partially positive hydrogen atoms of the methyl group. While classical (and still popular) force fields such as AMBER ignore these effects, FFLUX automatically captures them provided adequate training. All interactions between all atoms in a system are fed into the training. Unlike as in classical force fields, FFLUX does not adopt a ball-and-stick bonded/non-bonded architecture of penalty-based energy terms, even if some of them are coupled and expressed as cross-terms. Even in a small but pivotal molecule such as water FFLUX accounts [85] for a non-negligible (as much as 9%) three-body nature of bonded forces and angular forces.

## Data Availability

Not applicable.

## Footnotes

1 Names such atoms in molecules (AIM), energy decomposition analysis (EDA) or non-covalent interactions (NCI) are actually not helpful because they presumptuously claim that the method they refer to is the only one that covers what the name stands for. In the case of AIM, this has led to confusion when some researchers started (understandably though) using the acronym AIM to refer to non-AIM methods. This is why QTAIM is more precise and thus a decisively better name because it refers only to the original AIM. NEDA is also more specific and thus not suffering from the problematic acronym EDA. Hopefully NCI will also be replaced by a name that refers to that method only, rather than allow it to hog any alternative method to characterise non-covalent interactions.
